# Application of hybridized ensemble learning and equilibrium optimization in estimating damping ratios of municipal solid waste

**DOI:** 10.1038/s41598-024-67381-3

**Published:** 2024-07-30

**Authors:** Hossein Moradi Moghaddam, Mohsen Keramati, Alireza Bahrami, Ali Reza Ghanizadeh, Amir Tavana Amlashi, Haytham F. Isleem, Mohsen Navazani, Samer Dessouky

**Affiliations:** 1 School of Engineering, University of British Columbia, Kelowna, Canada; 2 Faculty of Civil Engineering, Shahrood University of Technology, Shahrood, Iran; 3 Department of Building Engineering, Energy Systems and Sustainability Science, Faculty of Engineering and Sustainable Development, University of Gävle, 801 76 Gävle, Sweden; 4 Department of Civil Engineering, Sirjan University of Technology, Sirjan, Iran; 5 School of Civil and Environmental Engineering and Construction Management, University of Texas at San Antonio, San Antonio, USA; 6 School of Applied Technologies, Qujing Normal University, Qujing, 655011 Yunnan China

**Keywords:** Municipal solid waste, Triaxial cyclic test, Damping, Ensemble learners, Machine learning, Equilibrium optimizer algorithm, Engineering, Civil engineering

## Abstract

The dynamic analysis of municipal solid waste (MSW) is essential for optimizing landfills and advancing sustainable development goals. Assessing damping ratio (D), a critical dynamic parameter, under laboratory conditions is costly and time-consuming, requiring specialized equipment and expertise. To streamline this process, this research leveraged several novel ensemble machine learning models integrated with the equilibrium optimizer algorithm (EOA) for the predictive analysis of damping characteristics. Data were gathered from 153 cyclic triaxial experiments on MSW, which examined the age, shear strain, weight, frequency, and percentage of plastic content. Analysis of a correlation heatmap indicated a significant dependence of D on shear strain within the collected MSW data. Subsequently, five advanced machine learning methods—adaptive boosting (AdaBoost), gradient boosting regression tree (GBRT), extreme gradient boosting (XGBoost), random forest (RF), and cubist regression—were employed to model D in landfill structures. Among these, the GBRT-EOA model demonstrated superior performance, with a coefficient of determination (R^2^) of 0.898, root mean square error of 1.659, mean absolute error of 1.194, mean absolute percentage error of 0.095, and an a20-index of 0.891 for the test data. A Shapley additive explanation analysis was conducted to validate these models further, revealing the relative contributions of each studied variable to the predicted D-MSW. This holistic approach not only enhances the understanding of MSW dynamics but also aids in the efficient design and management of landfill systems.

## Introduction

Annually, a significant volume of waste is produced worldwide, posing a major pollution challenge. According to a report by the World Bank, global waste generation is projected to increase to approximately 2.59 billion tons by 2030 and 3.4 billion tons by 2050^1^. About 40% of this waste is disposed of in landfills, considerably impacting the environment. The potential locations for constructing new landfills are increasingly limited due to stringent environmental regulations, rapid urbanization, and local opposition from residents near proposed sites^2^. Consequently, focusing on the design and development of sanitary landfills represents a pivotal step toward sustainable waste management. Landfills are exposed to a broad spectrum of failures under various loading conditions, as evidenced by incidents in OII (1994), Payatas (2000), Ano Liosia (2003), Leuwigajah (2005), and Shenzhen (2015). Studies of these failures reveal that multiple factors can precipitate such outcomes, including inadequate liner-waste contact, insufficient shear strength of the landfills, and other structural deficiencies^3^. Seismic events, in particular, can significantly compromise landfill integrity, leading to damage in the cover systems, disruption of leachate collection, and failures in gas collection systems. Therefore, municipal solid waste (MSW) components in landfills must be designed to remain secure and operational both during and after seismic activities to mitigate these risks. A thorough review of the literature shows that extensive research has been conducted to assess the behavior of MSW under dynamic loads on a laboratory scale. Notable examples include the cyclic investigations of MSW properties by Karim-pour Fard et al.^4^ and the study of MSW particle behavior concerning cyclic parameters by Rawat and Mohanty^5^. Damping ratio (D), a critical cyclic parameter, is the capacity of soil or similar materials to dissipate energy from vibrations or seismic waves. This property is pivotal in soil-structure interaction studies, particularly seismic response analysis. Understanding D is crucial as it provides valuable insights into how soils react under cyclic loads and assists in evaluating the stability and performance of geotechnical systems. The importance of D in earthquake design is underscored by numerous experimental studies. These include the use of a cyclic triaxial device to examine D of rubber particles mixed with clay by Akbarimehr and Fakharian^6^, the investigation of D in soft marine clay under cyclic shear stresses utilizing a cyclic simple shear device by Patino and Galindo^7^, and the study of D of clays across a wide range of shear strains from 0.001% to 1% employing a triaxial simple shear machine by Abdellaziz et al.^8^. It is crucial to acknowledge that the structure and performance of MSW can vary significantly across different regions due to variations in community consumption habits, weather conditions, and societal factors. For example, waste composition markedly differs between industrialized and developing countries^9^. Adopting computational methodologies can be a noticeable advancement in examining cyclic parameters, as it can overcome the limitations, high costs, and other challenges associated with laboratory and field evaluations. This approach enhances efficiency and provides a broader, more adaptable framework for understanding geotechnical behaviors under seismic conditions. Machine learning (ML) and artificial intelligence (AI) techniques are increasingly applied across various prediction problems, leading to the development of computational models based on empirical data^10^. Research in this field includes models for predicting the properties of MSW, such as the high heating quantity by Bagheri et al.^11^ and the biomass and waste properties by Liang et al.^12^. Additionally, Khatti and Grover^13^ assessed the compaction parameters of fine-grained soils using gene expression programming (GEP), least square support vector machine (LSSVM), long-short-term memory (LSTM), and artificial neural networks (ANNs). Comparative testing demonstrates that the linear LSSVM model MD110 and polynomial LSSVM model MD15 exhibited superior predictive capabilities. Ensemble ML models offer significant advantages over single models, primarily due to their improved prediction accuracy, robustness against overfitting, and enhanced generalization capabilities. By aggregating insights from multiple predictive models, ensembles leverage collective wisdom, which helps capture varied, subtle patterns within data that individual models might miss. This aggregation leads to better performance, as errors from individual models tend to cancel out, reducing bias and variance^14^. Moreover, ensembles are flexible and adaptable, capable of integrating different model types and structures to handle diverse data and relationships effectively. This makes them particularly effective in scenarios where the underlying data patterns are complex, and the cost of incorrect predictions is high^15^. Moradi Moghaddam et al.^16^ evaluated the shear modulus parameters of MSW and clay soil using various computational techniques, including random forest (RF), gradient boosting regression trees (GBRT), extreme gradient boosting (XGBoost), and adaptive boosting (AdaBoost). The study found that the GBRT methodology outperformed other methods in terms of model capabilities (R^2^, RMSE, MAE, and MAPE). Similarly, Ahmad et al.^17^ implemented an XGBoost model to predict the shear strength of rockfill materials. The performance of this model was assessed and compared with other predictive models such as AdaBoost, K-nearest neighbor, support vector machine, and random forest, with findings indicating that the XGBoost model presented the highest forecasting accuracy. Numerous studies have focused on various AI approaches to analyze the dynamic characteristics of all types of geomaterials, emphasizing the significance of these factors (Table 1). The DEEPSOIL program has been utilized to simulate landfill structures under various seismic loads, using dynamic parameters derived from numerical approaches^18,19^. The evaluation of numerical techniques in predicting the seismic properties of landfill components is an area of considerable scholarly interest, as it underscores the application of ML in the geo-environmental sector. This approach not only saves time and money in laboratory and field tests but also addresses the notable scarcity of data regarding the cyclic properties of MSW. Consequently, this article focuses on developing a reliable method for calculating D, an emerging topic that promises to enrich our understanding of seismic engineering in landfills. Drawing from previous studies that display the high accuracy of ML techniques in estimating MSW properties, this research employs ensemble-based methods to predict D parameter of landfill MSW components. Initially, the study determines factors influencing D-MSW through a cyclic triaxial device. Subsequently, prediction models for D in landfill components will be developed using various methods, including GBRT, XGBoost, RF, AdaBoost, and Cubist, which are then hybridized with the equilibrium optimization algorithm (EOA). EOA is a robust optimization method inspired by the thermodynamic principle of mass balance, effectively balancing exploration and exploitation to efficiently find optimal solutions in complex problem spaces^20^. This makes it especially adept at dealing with nonlinear, multimodal, and high-dimensional challenges where traditional methods may falter. The EOA’s adaptability and simplicity, with fewer parameters to manage and no need for complex operations like those in genetic algorithms (GAs) or particle swarm optimization (PSO), enhance its appeal^21^. In predictive modeling, such as for D in seismic analysis, EOA can remarkably improve ensemble models by optimizing model parameters and combining predictions effectively, leading to more accurate and reliable outputs^22^. This adaptability and efficiency make EOA an attractive choice for complex optimization tasks, ensuring robustness and superior performance across various applications^23^.

**Table 1:** Study overview of ML utilization in D estimation.

References	Year	Materials	AI Methods
Moradi Moghadam et al.^16^	2024	MSW and clay soil	AdaBoost, GBRT, XGBoost, and RF
Gatto and Montrasio^25^	2023	Polyurethane-sand combination	Feed forward neural network (FNN)
Wu et al.^26^	2023	Marine clay	Back-propagation neural network (BPNN)
Baghbani et al.^27^	2023	Dilative silica sand	Classification and regression random forest (CRRF) and ANN
Baghbani et al.^28^	2023	Dry sand	ANN and support vector machine (SVM)
Pasha et al.^29^	2020	Tire-gravel chips combination	Support vector regression (SVR) and ANN
Keshavarz and Mehramiri^30^	2015	Sand	GEP
Javdanian et al.^31^	2015	Fine grained soils	ANN and ANFIS
Edincliler et al.^32^	2013	Waste-sand tires combination	Neural networks (NN) and neuro–fuzzy (NF)
Samui and Kothari^33^	2012	Synthetic reinforced soil	Multivariate adaptive regression spline (MARS), adaptive neuro-fuzzy inference system (ANFIS), multilayer perceptron (MLP), and multiple regression analysis method (MRM)
Cevik and Cabalar^34^	2009	Mica-sand combination	GEP
Cabalar and Cevik^35^	2009	Mica-sand combination	NN
Akbulut et al.^36^	2004	Reinforced sands	ANFIS, MLP, and MRM

Proposed models in this research offer geotechnical engineers vital tools for enhancing the seismic resilience of landfills. These models are capable of predicting the dynamic properties of MSW across a variety of conditions, facilitating precise and informed decision-making in engineering practices. Utilizing Shapley additive explanation (SHAP) values, the models deliver clear, interpretable insights into how various factors such as the age, shear strain, and material composition influence dynamic behavior^16^. This detailed analysis helps engineers prioritize specific design and operational adjustments, like optimizing compaction methods or selecting appropriate materials aimed at bolstering seismic stability. In essence, these models convert complex dataset analyses into practical, actionable guidance that significantly improves landfill design and management, contributing to enhanced safety and resilience against seismic activities^24^.

## Experimental framework

### Characteristics of Kahrizak landfill site

**Figure 1:**
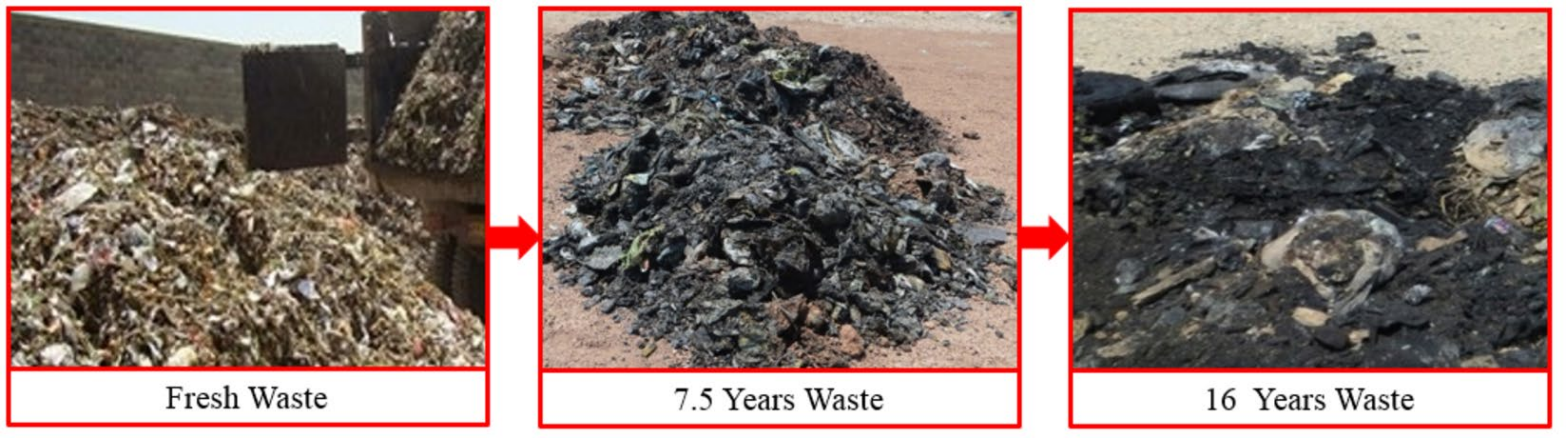
Various MSW samples.

Kahrizak landfill site (KLS), with an area of about 15 km^2^, is the largest landfill in the Middle East. About 8000 tons of waste per day enter from Tehran province to this center. Based on the samples of waste collected for ingredient study, organic material comprised most of the components across a wide range of particle dimensions^37^. KLS is located in a vulnerable ecological area, and the landfill’s activities have resulted in environmental deterioration, groundwater and soil pollution, and considerable health problems for surrounding populations. Figure 1 depicts a broad picture of various MSW samples at ages of fresh, 7.5 years, and 16 years.

### Experimental assessments of D-MSW

In this section, three types of MSW specimens are recognized and gathered: fresh, 7.5 years, and 16 years. The aging process in waste samples causes significant chemical and physical modifications in the composition. The examination of the variations in waste elements over various ages reveals that as the age of trash grows up to 16 years, the proportion of moisture content and organic materials drops (193% and 188%, respectively), while the amount of plastic components rises (about 160%) (Table 2).

**Table 2:** MSW components at different ages.

Components	Percentage of components		
	16 years	7.5 years	Fresh
Organic waste	21.7	39.8	62.6
Paper	8	9.4	14.6
Plastic	44.6	32.2	17.1
Glass	9.6	5.2	2.4
Metal	0.8	1.6	1.8
Wood	3.4	2.4	0.6
Soil and rock	11.9	9.4	0.9
Total	100	100	100

**Figure 2:**
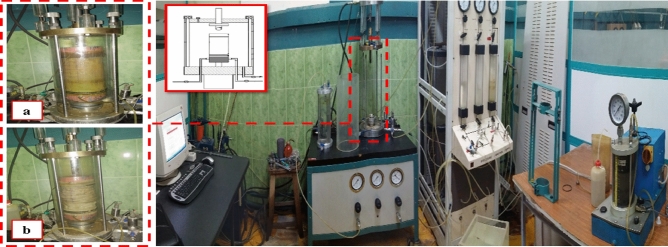
Real and schematic images of cyclic triaxial apparatus and samples: (**a**) before testing, (**b**) after testing.

D-MSW was determined through experiments employing a strain-controlled cyclic triaxial machine (Fig. 2) and consolidated undrained compressive tests. The experimental setup included two pore water pressure sensors (accuracy of 0.2 kPa and measurement range of 0–1000 kPa), one axial strain sensor (accuracy of ± 0.001 mm and a measurement range of 70 mm (horizontal)), and one load cell (precision of 0.0025 kN and a measurement range of ± 20 kN). The cyclic triaxial apparatus utilized for testing could accommodate samples with a height of 20 cm and a diameter of 10 cm, allowing the assessment of dynamic properties within the shear strain (varies from 0.08% to 4%). The selection of the maximum particle dimension was based on recommendations from Zekkos et al.^38^, specifying that the most significant size might be up to one-third of the device’s sample size. Notably, ASTM D4767 and ASTM D3999 standards were applied during the loading and analyzing phases, respectively, considering the distinct characteristics of MSW.

**Figure 3:**
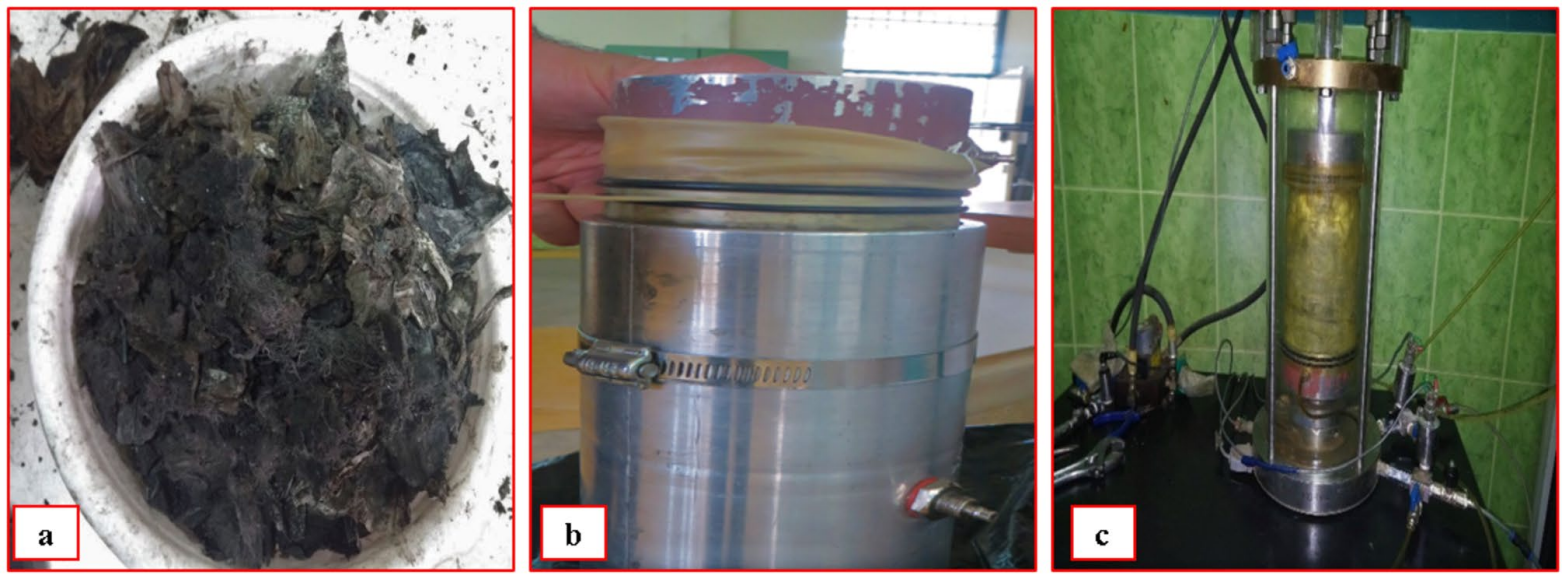
Reconstruction of triaxial sample.

To empirically assess the shear modulus parameter, 153 cyclic triaxial tests were conducted on waste specimens. These tests covered various factors such as the age (ranging from 0 to 16 years), plastic content (ranging from 0 to 0.274), confining pressures at two values of 75 kPa and 150 kPa (chosen to reflect conditions at KLS with a maximum waste height of 10 m to 20 m), unit weights of 9 kN/m^3^and 12 kN/m^3^ (corresponding to nearly observed unit weights for a fresh and aged sample of wastes in the ranges of 7 kN/m^3^ to 10 kN/m^3^ and 11  kN/m^3^ to 13 kN/m^3^), and frequencies of 0.1 Hz, 0.5 Hz, and 1 Hz, and shear strain levels of 0.075, 0.148, 0.37, 0.735, 1.83, and 3.63. The cyclic triaxial tests were conducted on reconstructed samples under consolidated undrained (CU) levels. Fresh and aged samples reconstitution adhered to the guidelines outlined in ASTM D4767. Following the reconstitution of the specimens and the setup of the apparatus, saturation of the specimen was achieved by ASTM D4767-95. The attainment of B values greater than 0.9 confirmed the saturation of the sample. During the consolidation stage, the cell pressure was incrementally raised. The initiation of the consolidation phase occurred upon opening the drainage valve, marking the point when the sample had attained the required confining stress. This consolidation phase persisted until volume variation ceased, and any excess pore water pressure completely dissipated, reaching equilibrium with the back pressure. Notably, due to the low permeability of MSW, the consolidation stage extended beyond 10 h. Following the consolidation period, the drainage valve was closed to fulfill the requirements of consolidated undrained conditions for subsequent cyclic stress applications. The primary phases of reconstructing the samples involved: (**a**) separation of the MSW samples, (**b**) installation of the membrane and mold along with the placement of the paper filter and upper cap, and (**c**) placement of the samples (Fig. 3).

## Methodology and data description

Choosing between black-box, grey-box, and white-box ML models often hinges on the trade-off between  the accuracy and interpretability, which is crucial in the fields like engineering and environmental science. Black-box models like ensemble learners typically provide high predictive accuracy by capturing complex nonlinear relationships within large, high-dimensional datasets. However, their opaque decision-making processes limit their interpretability^39^. In contrast, grey-box models offer a middle ground by incorporating both theoretical knowledge and data-driven elements, enhancing transparency and user trust without completely sacrificing performance. Meanwhile, white-box models like linear regression and decision trees prioritize transparency, with straightforward, understandable decision pathways that make them ideal for educational purposes and settings where understanding the causality behind predictions is paramount^39^. However, their simplicity can be a drawback when facing complex interactions that require more nuanced or intricate modeling. Ultimately, the choice among these model types should be guided by the specific requirements and constraints of the application at hand, weighing the need for accuracy against the importance of transparency and the ability to understand and trust the underlying mechanisms driving predictions^39^. In the following, we introduce the models implemented in this study in more detail and compare their mechanisms for modeling more accurately.

### AdaBoost

**Figure 4:**
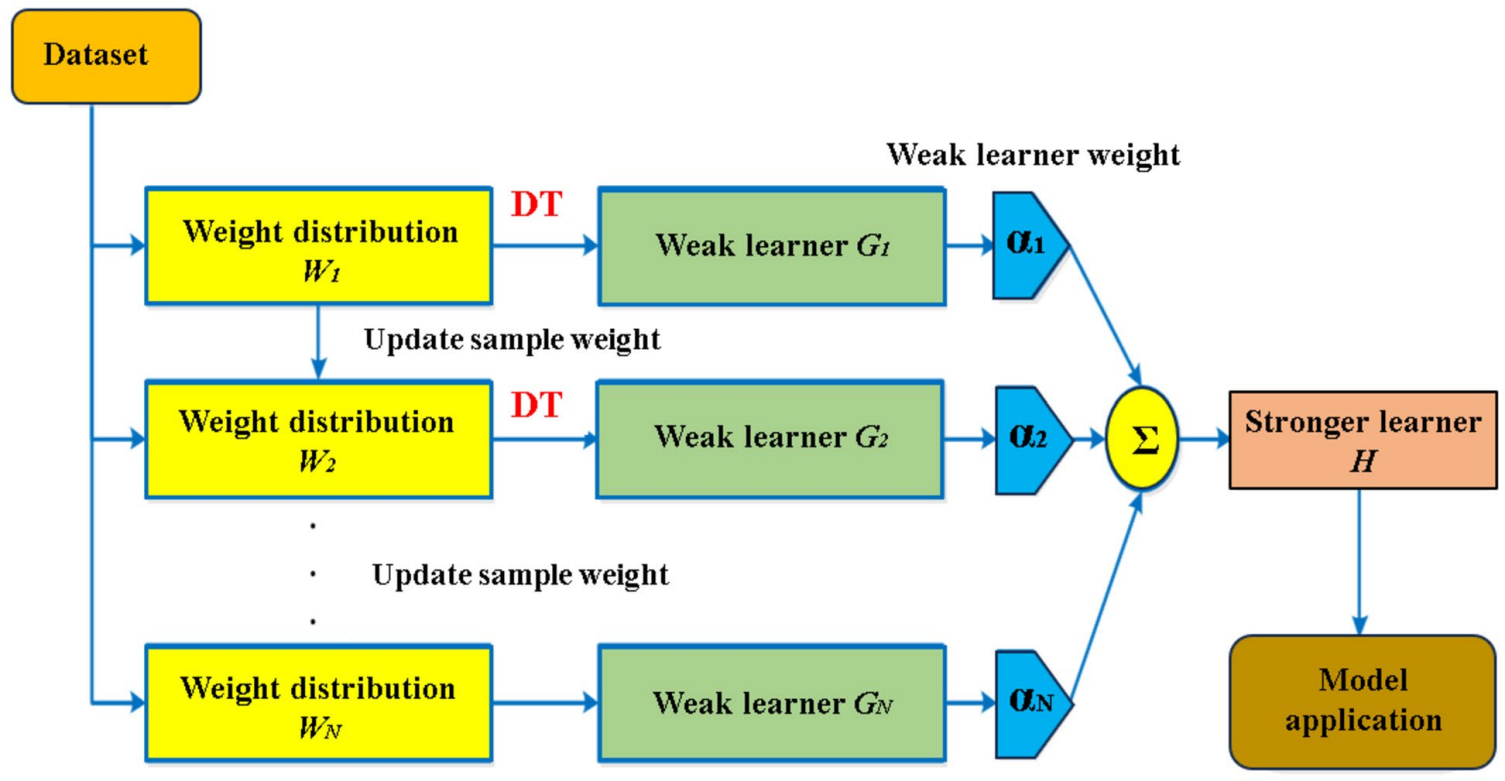
Sequence of steps in AdaBoost modeling process.

The principle behind the AdaBoost method is to appropriately integrate numerous weak classifiers to create a strong one^40^. AdaBoost employs iteration and trains just a single weak classifier for each cycle. The learned weak classifier will be included in the following iteration. As a result, after the *N*_*th*_ iteration, there will be the entire *N* weak classifiers, *N−1* of which has already been trained, and its different variables will remain unchanged. Currently, the N_th_ classifier is being trained. The link among the weak classifiers is that the N_th_ weak classifier is more likely to categorize the data than the initial *N−1* weak classifiers that do not classify. The ultimate categorization result is determined by the total impact of the *N* classifiers. Figure 4 provides an overview of the AdaBoost model.

### GBRT

**Figure 5:**
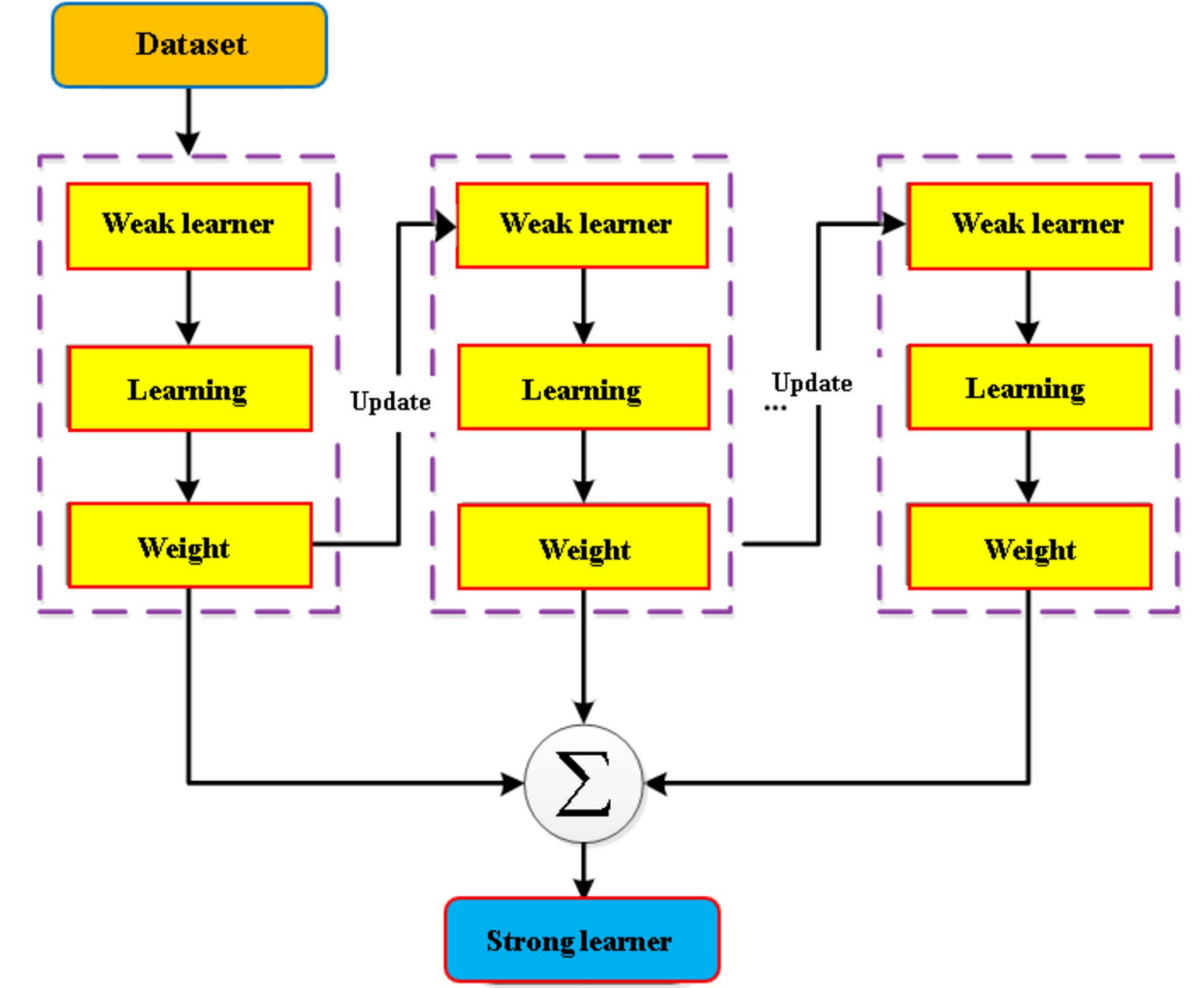
Basic process of GBRT.

GBRT is an example of integrated learning boosting that employs a forward-distributing technique^41^. This technique seeks to minimize the loss function by picking a suitable tree function according to the present theory and fitting function; hence, GBRT comprises two sections: the gradient boosting and regression tree. A regression tree is utilized to forecast the actual amount. Gradient boosting iterates across many trees to jointly determine the outcome. Each tree is the culmination and remnant of the previously learned trees. Figure 5 provides an overview of the GBRT model.

### XGBoost

**Figure 6:**
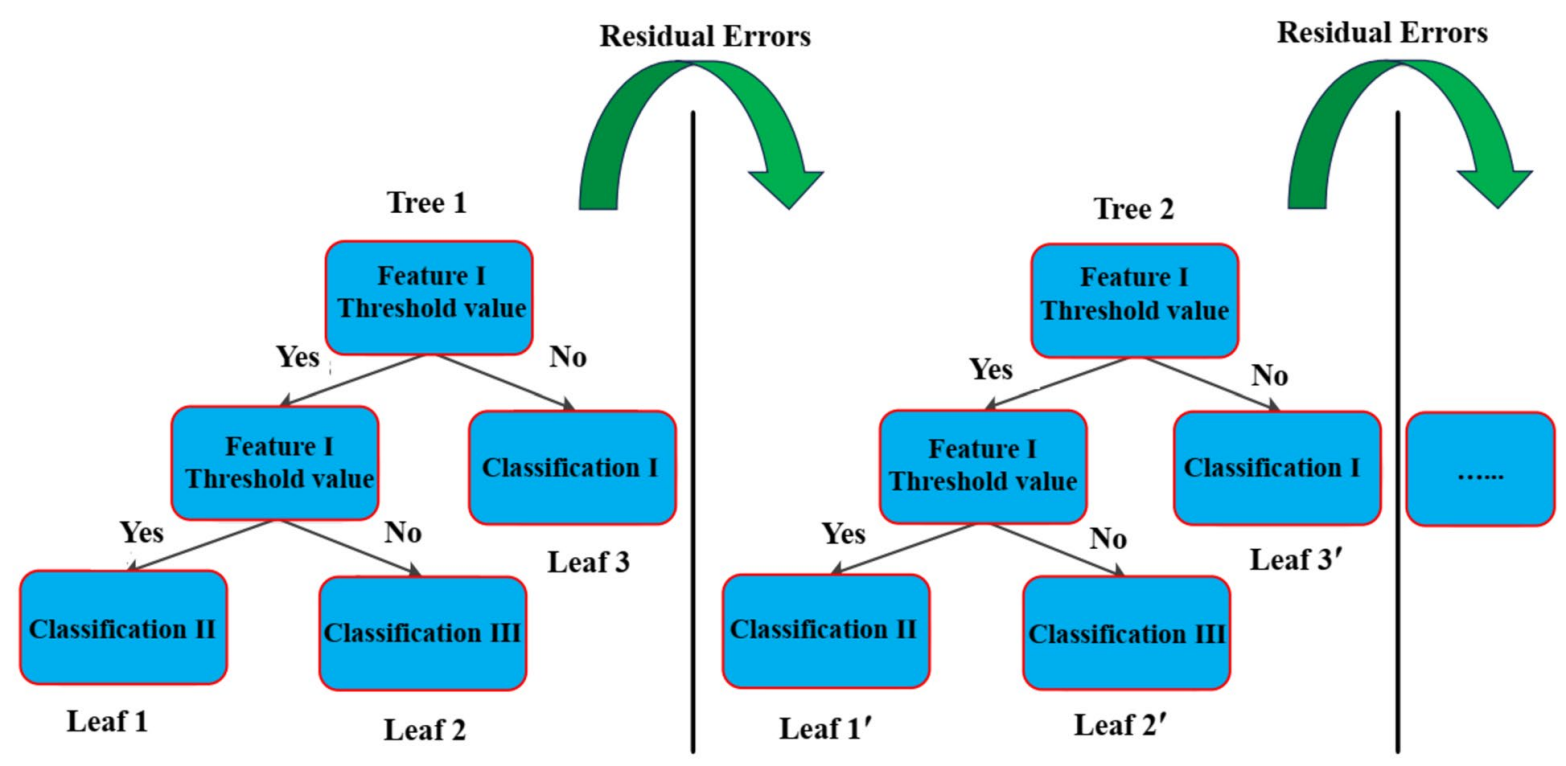
A flowchart illustrating structure of XGBoost trees.

XGBoost is a popular software package that utilizes gradient boosting methods for supervised education. Its excellent performance, versatility, and capacity make it a popular choice for ML contests and commercial use^42^. XGBoost supports a wide range of data formats, such as numerical and qualitative characteristics, and it contains sophisticated functions like early ending and tree removal to increase model generalization and training rate^43^. The XGBoost flexibility and resilience provide an excellent choice for organized and formatted data evaluation. XGBoost deploys a mixed training approach in which fundamental designs are included in a forward-step fashion. The XGBoost algorithm is defined by Fig. 6.

### RF

**Figure 7:**
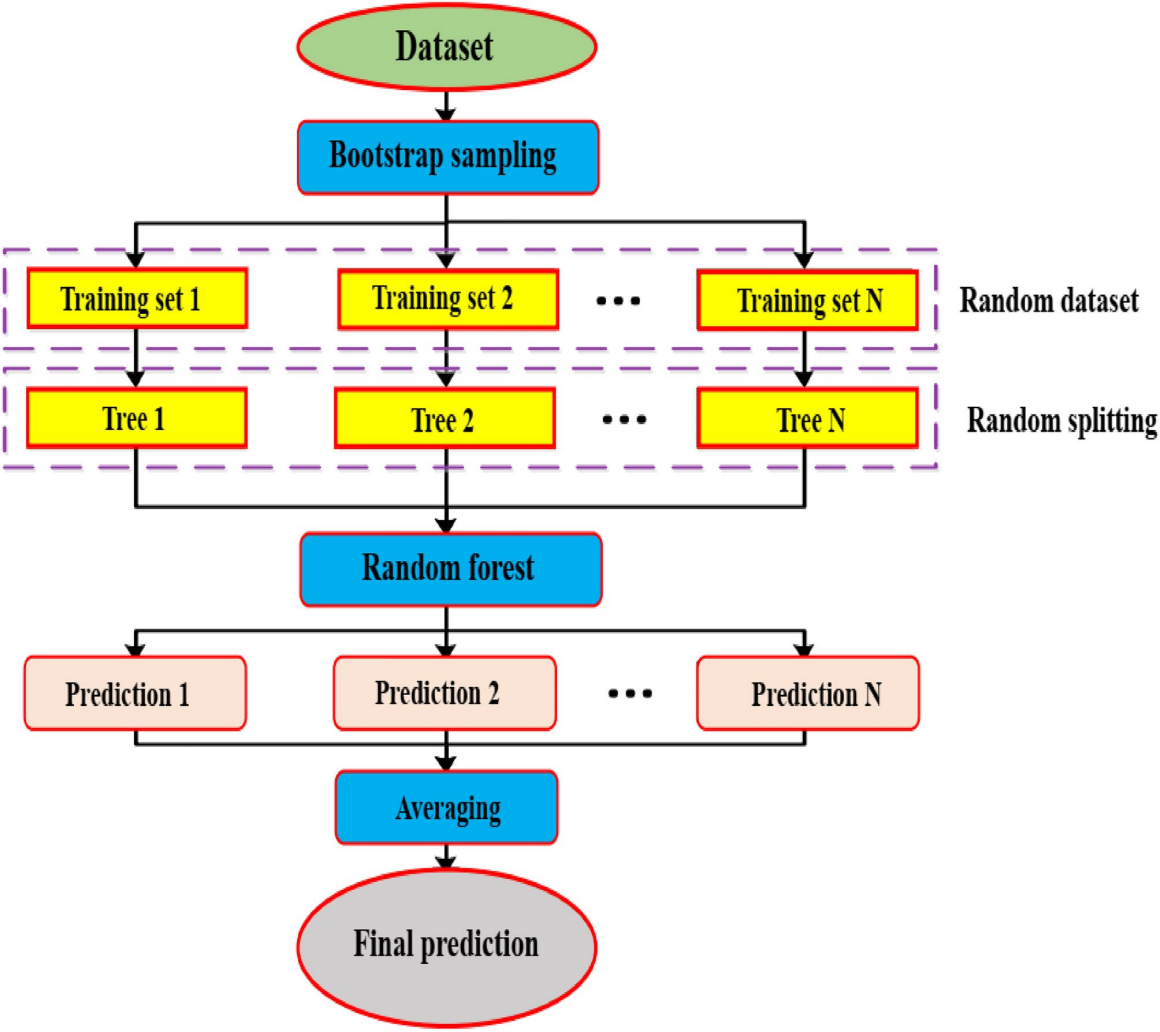
Schematic flowchart of RF.

Svetnik introduced the model of RF regression as an enhanced regression approach with classification capabilities^44,45^. RF comprises several decision trees, therefore, there is no linkage among them. By conducting classification work with a new study sample, each decision tree within the forest is evaluated and categorized independently. Following that, every decision tree will receive a categorization result (Fig. 7). The decision tree with the most classification outcomes will be the outcome^46^.

### Cubist

**Figure 8:**
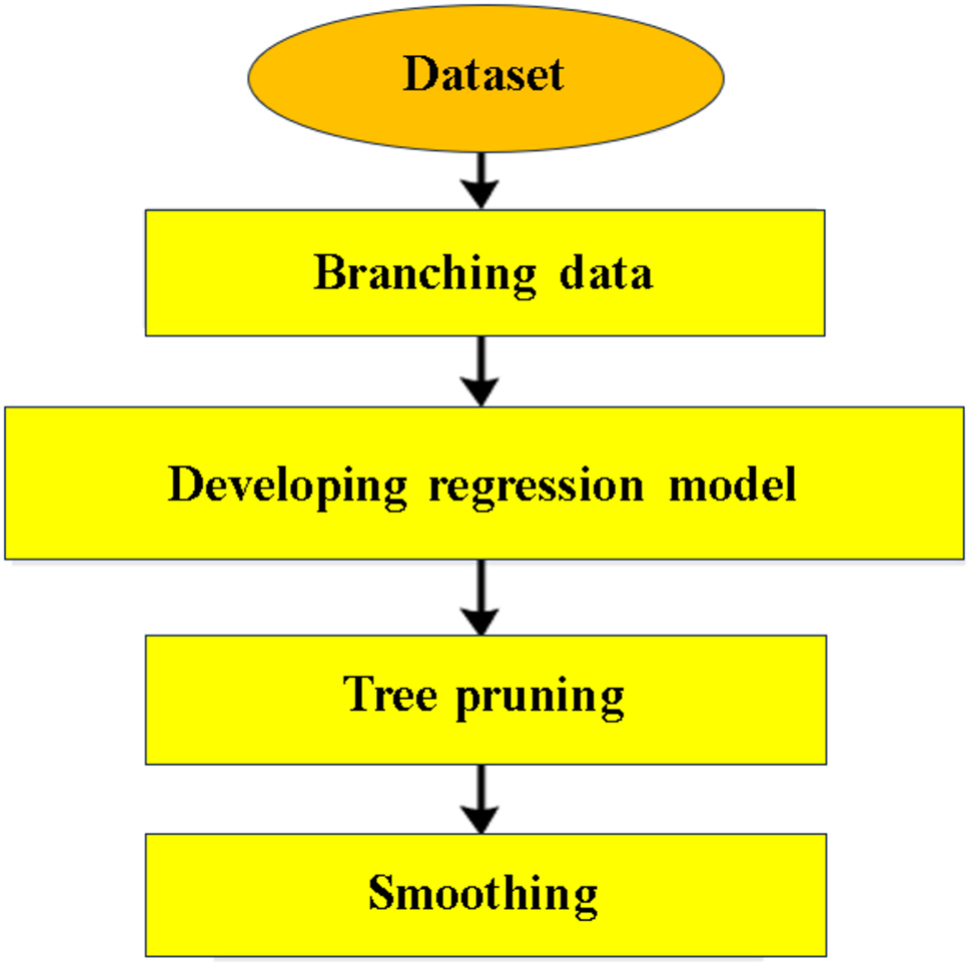
Flow diagram of Cubist model.

Using Quinlan’s M5 model tree^47^, Cubist as a non-parametric ML model^48^ constructs predictive models with the capability of handling up to thousands of input variables^49^. Wang and Witten^50^ improved the M5 tree with better performance. In a Cubist model, predictions are created with nodes different from a regression tree. In a regression tree, each node is assigned a single value prediction, while in the Cubist model, each node is assigned a linear regression model prediction^51^. By combining two models linearly, Cubist models can balance their weights better. Multiple training committees and reinforcement are included in this approach^52^. Final predictions are produced by averaging predictions from the committees^53^. Several hyper-parameters (for instance number of rules, number of committees, number of neighbors, and exploration constant) must be optimized to improve the model performance. A smoothing process can determine the number of nodes. Furthermore, using boosting^54^, multiple ensemble models can be generated by generating several committees. Figure 8 shows the flow diagram of the Cubist model.

### EOA

Faramarzi et al.^20^ were the first to provide a dynamic mass balance-inspired EOA. Utilizing multiple sources and sink processes, EOA methods determine the concentration of an element in a control volume. According to basic mechanics, the entrance, departure, and generation of mass are retained in the equation for mass equilibrium. A crucial component of traditional EOA is the iterative update of the beginning amounts of particles with specified numbers^55^. These solutions are extracted as the top possibilities to identify the equilibrium state regarded as the desired result. Based on an objective function, particle concentrations are assessed repeatedly to determine their states. The four best particles, arithmetic means, and the equilibrium pool are considered seriously by the EOA updating process as follows:

1 $${X}_{new}={X}_{eq}+\frac{G}{\lambda }\left(1-F\right)+\left(X-{X}_{eq}\right)\cdot F$$

where *X*_*new*_ and *X* represent the particle’s new and current concentration vectors, a random concentration vector called *X*_*eq*_ will be chosen from the equilibrium pool. *F* is an exponential term described in Eq. (2); *G* is the generation rate defined in Eq. (3), and *λ* is a random vector between 0 and 1.

2 $$F={a}_{1}sign\left(m-0.5\right).({e}^{-\lambda \left(1-\frac{T}{{T}_{max}}\right){a}_{2}T/{T}_{max}}-1)$$

3 $$G=\left\{\begin{array}{c}0.5{r}_{1}\left({X}_{eq}-\lambda X\right)F if {r}_{2}\ge GP\\ 0 if {r}_{2}<GP\end{array}\right\}$$

**Figure 9:**
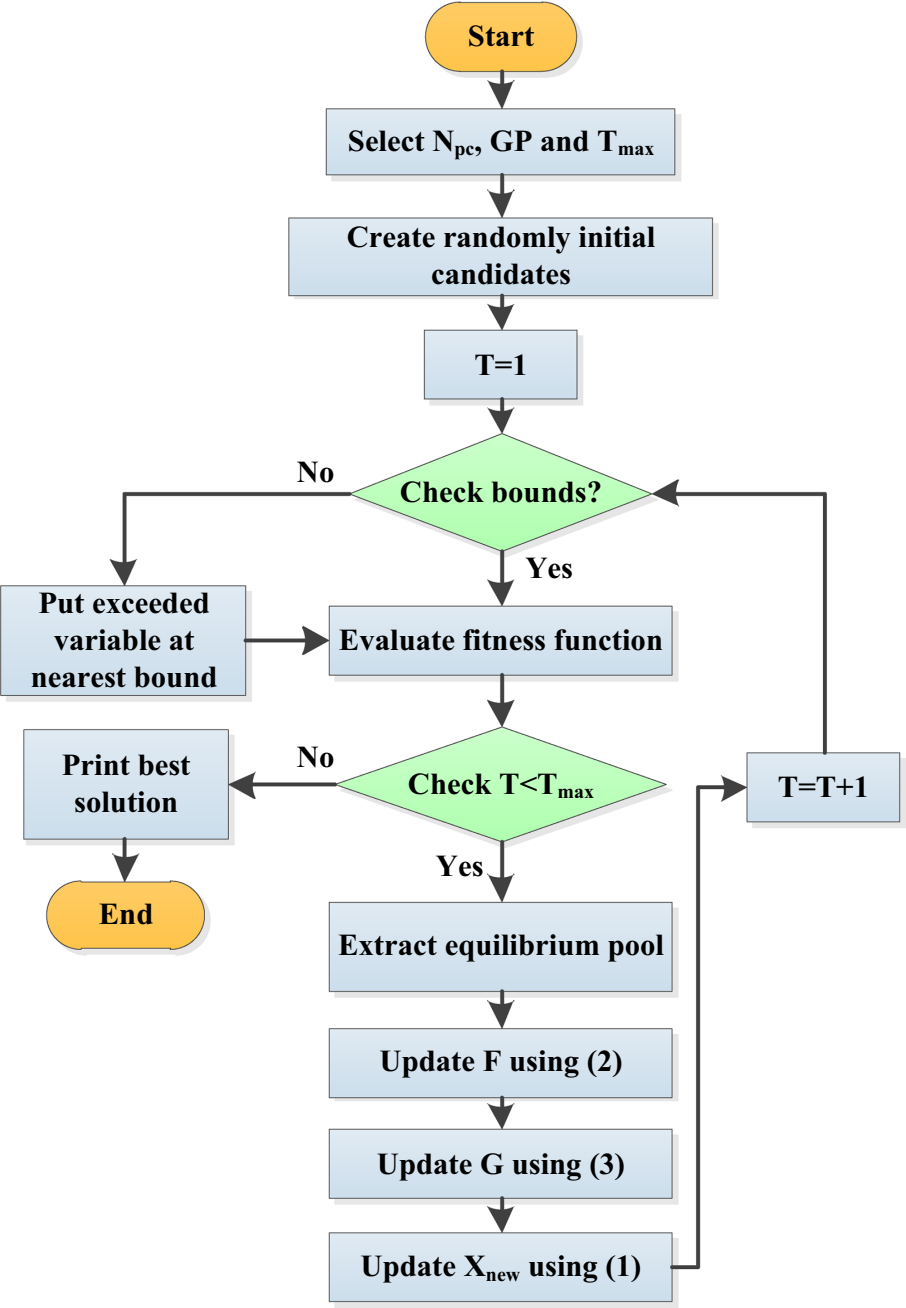
General steps of conventional EOA.

Here, *m* is a random vector between 0 and 1, *a*_*1*_ and *a*_*2*_ are constants (*a*_*1*_ = 2 and *a*_*2*_ = 1), while *T* and *T*_*max*_ are the present and maximum iterations. *r*_*1*_ and *r*_*2*_ are two distinct random variables, and *GP* is a specific value known as the generation probability (*GP* = *0.5*). According to the three components in Eq. (1), the updating process of each concentration particle is renewed. In these latter two sections, the search area is effectively utilized, and a global search is carried out to identify the ideal outcome^55^. Figure 9 demonstrates the steps of the standard EOA.

### Implementation process

#### Data collection

**Figure 10:**
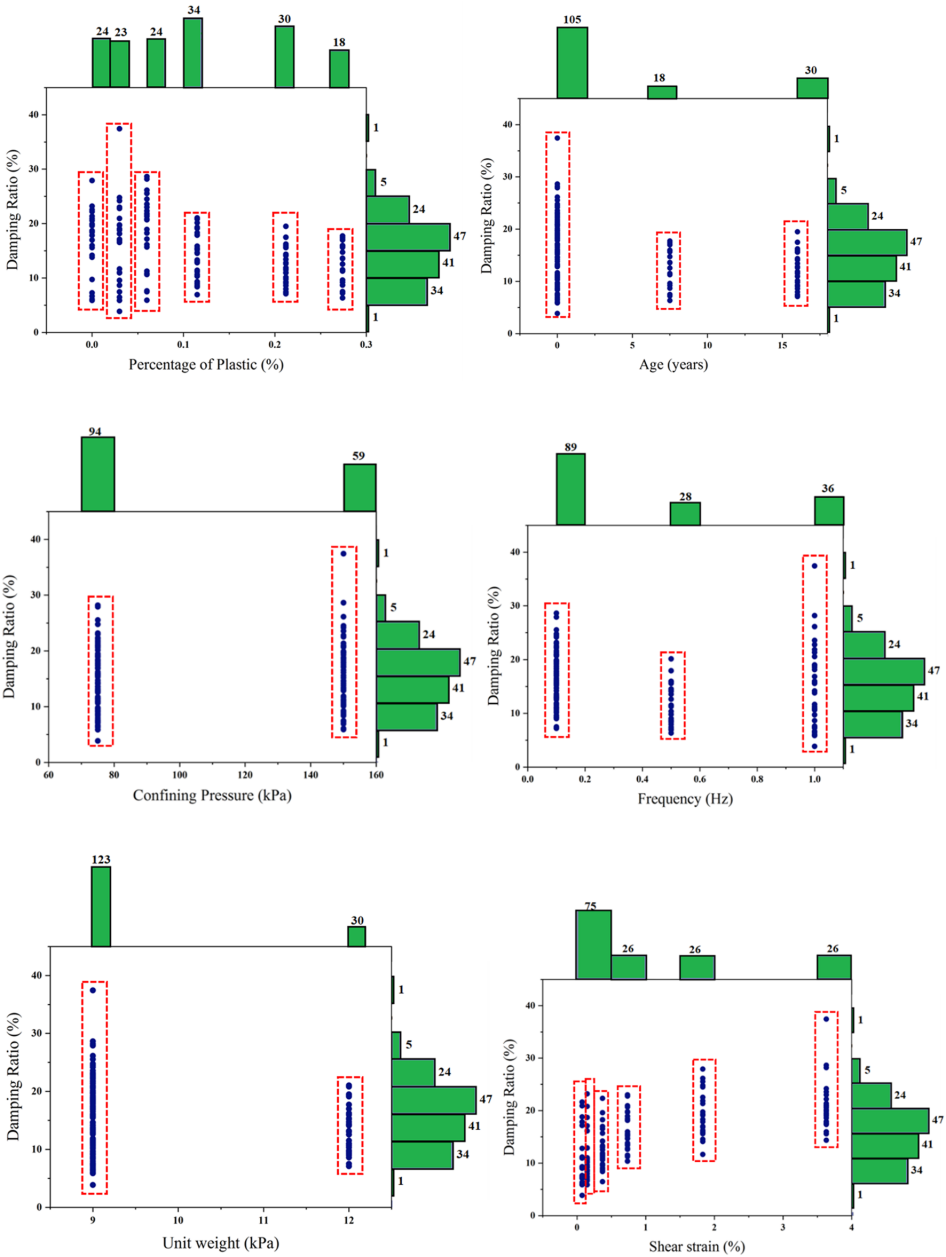
Pairwise correlation between dependent and independent factors of D-MSW database.

**Figure 11:**
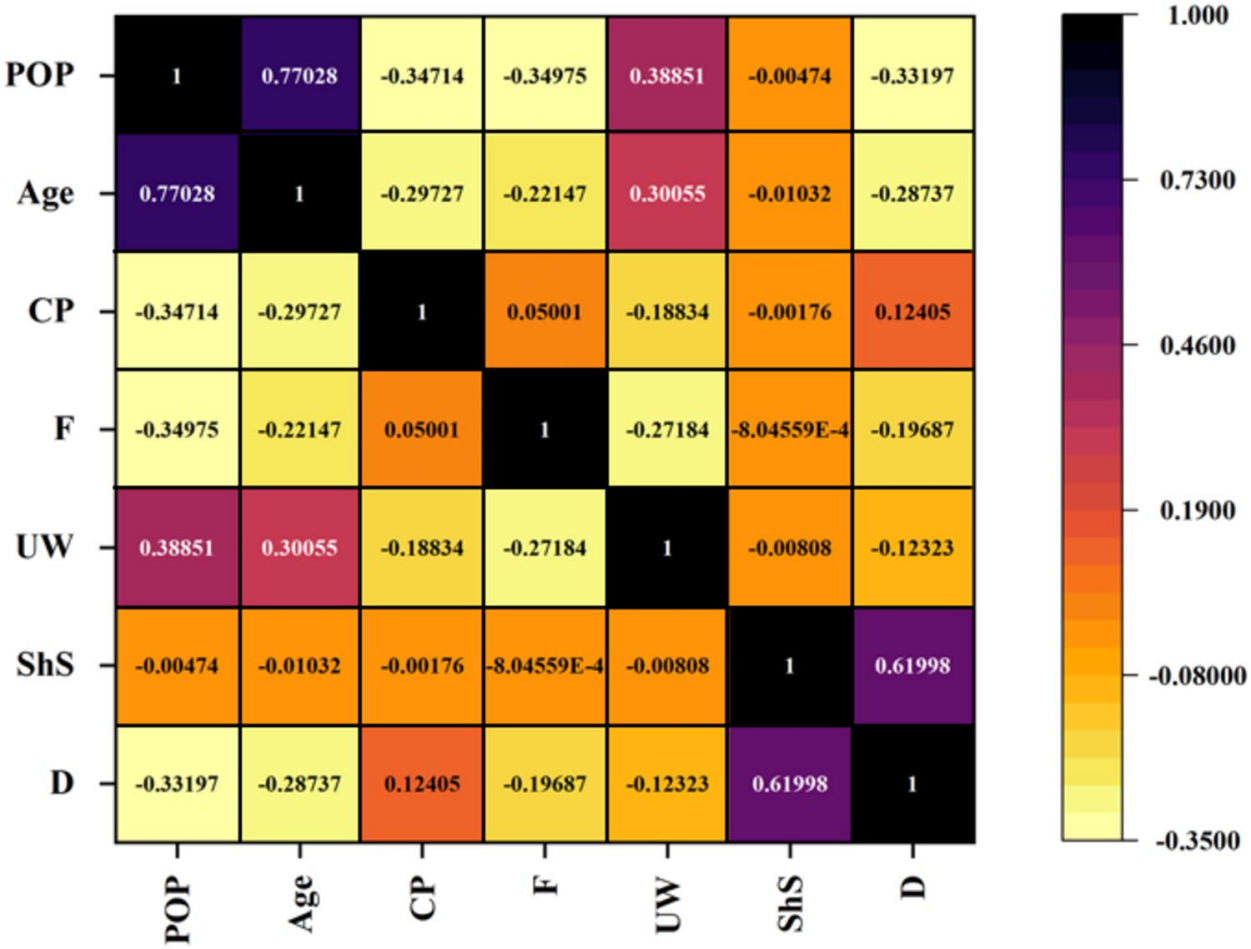
Pearson coefficients of factors engaged in estimation of D-MSW.

The database utilized in this study consists of 154 data records for waste D, which were obtained from sampling, making specimens, and performing dynamic tests in the laboratory as described above. Additionally, in this study, six variables, including the age of MSW (*Age*), frequency (*F*), percentage of plastic (*PoP*), confining pressure (*CP*), shear strain (*ShS*), and unit weight (*UW*), for D-MSW were considered as inputs. Figure 10 exhibits pairwise correlations between independent and dependent variables, including the frequency histogram of each variable. The graph displays a non-uniform distribution of outputs, and the factors are appropriately more frequent. High-frequency factors can be used for a better model^56^. A lower correlation is observed between positive and negative quantities in the general model variable, as indicated in Fig. 11. Also, each of the selected variables adversely affects D. The correlation heatmap analysis reveals the greater dependence of D on shear strain in the MSW database. Meanwhile, the percentage of plastic has the lowest correlation with output in D databases.

Modeling began with randomly dividing the data into training and testing subsets. To accomplish this, 70% of the data were randomly chosen as train data and 30% as testing data. Table 2 presents the statistical characteristics of output and input variables for test and train data for D-MSW. According to Table 3, the value of *PoP* is between 0 and 0.274 percent, *Age* varies between 0 and 16, *CP* falls between 75 and 150, *F* has two values of 0.1 and 1, *UW* varies from 9 to 12, and *ShS* ranges from 0.075 to 3.63 for D-MSW. Also, the measured D in this database changes in the range of 5.89 and 37.45 percent for training and the range of 3.89 and 24.21 percent for testing. Besides, various ranges of output changes in each output depict that the databases are diverse, and the models developed over them are generalizable^57^.

**Table 3:** Comprehensive statistics for both test and train data related to D-MSW.

Statistic	*PoP*	*Age*	*CP*	*F*	*UW*	*ShS*	D
Train data: 107							
Minimum	0	0	75	0.1	9	0.075	5.89
Maximum	0.274	16	150	1	12	3.63	37.45
Mean	0.109	3.911	103.738	0.39	9.505	1.213	15.445
Median	0.115	0	75	0.1	9	0.735	15.612
Standard deviation	0.091	6.412	36.634	0.378	1.127	1.307	6.014
Variance	0.008	41.112	1342.025	0.143	1.271	1.709	36.164
Skewenss	0.514	1.208	0.487	0.79	1.799	0.99	0.61
Kurtosis	− 1.092	− 0.323	− 1.796	− 1.101	1.26	− 0.544	0.538
Test data: 46							
Minimum	0	0	75	0.1	9	0.075	3.89
Maximum	0.274	16	150	1	12	3.63	24.21
Mean	0.124	4.272	104.348	0.374	9.783	1.01	14.259
Median	0.115	0	75	0.1	9	0.735	14.392
Standard deviation	0.095	6.426	37.008	0.368	1.332	1.178	5.226
Variance	0.009	41.297	1369.565	0.135	1.774	1.388	27.307
Skewenss	0.282	1.087	0.461	0.895	1.126	1.392	− 0.017
Kurtosis	− 1.257	− 0.515	− 1.871	− 0.866	− 0.767	0.716	− 0.986

#### Model performance evaluation criteria

Selecting appropriate error measures like R^2^, RMSE, MAE, MAPE, objective function (OBJ), and a20-index for evaluating the accuracy and reliability of models in this study was driven by their specific relevance to the objectives and data characteristics^22,58^. The R^2^ metric, or coefficient of determination, is crucial as it determines the proportion of variance in the dependent variable that is predictable from the independent variables; an R^2^ value close to 1 is ideal, indicating a model that accurately predicts the observed outcomes, whereas a value near zero suggests the model fails to predict the data effectively. RMSE, provides insights into the average model prediction errors with sensitivity to outliers, meaning it measures the magnitude of errors with more significant discrepancies having a greater impact on the RMSE value^59^. An ideal RMSE value is as low as possible (zero), exhibiting more minor prediction errors. MAE, measures the average magnitude of the errors without considering their direction, being less sensitive to outliers compared to RMSE and thus offering a straightforward average of absolute differences between predicted and actual values. Lower MAE values signify the improved model performance, with the ideal value being as close to zero as possible. MAPE, expresses prediction errors as a percentage of actual values, making it particularly useful for comparisons across different scales or units. Lower MAPE values (ideal being close to zero) represent more accurate models. The OBJ function combines several statistical measures to provide a comprehensive summary of model errors, encompassing components like bias, variance, and overall fit. An ideal OBJ value is minimized, reflecting balanced and low error metrics^22^. The a20-index offers a unique perspective by focusing on the accuracy within the 20th percentile of predictions. It is crucial for scenarios where smaller prediction errors are critical, as it highlights the model’s performance in accurately predicting the lower range of data values. A higher a20-index value is ideal, demonstrating better performance in this context^23^. Each of these metrics was chosen to ensure a holistic evaluation of the model’s performance, directly supporting the study’s aims to develop reliable predictive models for real-world applications, thereby providing a comprehensive assessment of the model’s accuracy, robustness, and practical utility. The performance statistical metrics are illustrated as follows:

4 $$R^{2} = \left[ {\frac{{\sum\limits_{i = 1}^{N} {(Y_{obs} - \overline{Y}_{obs} )(Y_{pre} - \overline{Y}_{pre} )} }}{{\sqrt {\sum\limits_{i = 1}^{N} {(Y_{obs} - \overline{Y}_{obs} )^{2} } \sum\limits_{i = 1}^{N} {(Y_{pre} - \overline{Y}_{pre} )^{2} } } }}} \right]^{2}$$

5 $$RMSE = \sqrt {\frac{1}{N}\sum\limits_{i = 1}^{N} {(Y_{pre} - Y_{obs} } )^{2} }$$

6 $$MAE = \frac{{\sum\nolimits_{i = 1}^{N} {\left| {Y_{pre} - Y_{obs} } \right|} }}{N}$$

7 $$MAPE = \frac{{\sum\nolimits_{i = 1}^{N} {\left| {Y_{pre} - Y_{obs} } \right|} }}{{\sum\nolimits_{i = 1}^{N} {Y_{obs} } }} \times 100$$

8 $$OBJ = \left(\frac{{N_{tr} }}{{N_{all} }}.\frac{{RMSE_{tr} + MAE_{tr} }}{{R_{tr}^{2} + 1}}\right) + \left(\frac{{N_{tst} }}{{N_{all} }}.\frac{{RMSE_{tst} + MAE_{tst} }}{{R_{tst}^{2} + 1}}\right)$$

9 $$a20 - index = \frac{m20}{N}$$

When *N* is the number of records, *Y*_*pre*_ and *Y*_*obs*_ present the predicted and actual values, and the bar items over the parameters show the average rate; The variable *m20* displays the quantity of the records where the *Y*_*obs*_*/Y*_*pre*_ ratio ranges from 0.80 to 1.20; the terms "*tst*" applied for testing and "*tr*" applied for training data, accordingly.

#### Proposed hybrid EL models

**Figure 12:**
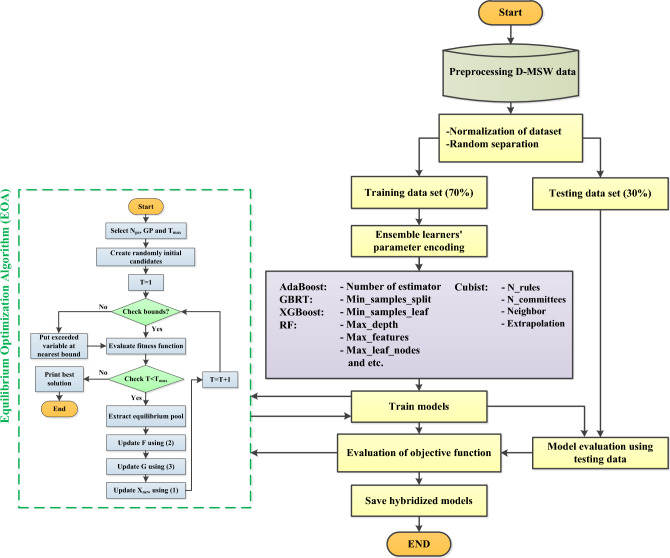
An overview of implementation process of EL-EOA methods.

The suggested ML models in the present research were all constructed in Python. EOA was employed in this study to determine the ideal values. They utilized the given criteria to set the initial random values to accomplish this (Table 4). After these statistics were entered into EL approaches and the EL algorithms were trained using the training dataset, the target function was determined to be the average RMSE of both data (test and train). The meta-parameter volume and objective function quantities are sent into EOA to optimize. Figure 12 provides a summary of the various EL approaches. Table 5 lists the optimal values of numerous meta-parameters using different approaches for the D-MSW model.

**Table 4:** Various ranges of parameters are considered for optimization.

Parameters	Applied range	Parameters	Applied range
Number of estimators	[5,200]	Number of committees	[2,5]
Min_samples_split	RF: [1,10] Other models: [1e−10,1]	Neighbors	[1,9]
Min_samples _leaf	RF: [1,10] Other models: [1e−10,1]	Extrapolation	[1e−5,0.5]
Max_depth	[2,500]	lu_ns	[2,150]
Max_features	[1, maximum value of variables]	lu_max_d	[2,100]
Max_ leaf_nodes	[2,500]	lu_max_mlf	[2,100]
Ccp_alpha	[0,1]	lu_lr	[0.0001,1]
Min_weight_fraction_leaf	[0,0.5]	lu_gamma	[0,10]
Learning rate	[0.001,3]	lu_gamma	[0,10]
Alpha	[0.001,0.99]	lu_min_cw	[0,1]
Subsample	[1e−6,1]	lu_subsample	[0.5,1]
Max_samples	[0.1,1]	lu_subsample_bt	[0.5,1]
Number of rules	[1000,10,000]	reg_lambda	[0.01,2]

**Table 5:** Optimized values of meta-parameters for D-MSW.

Parameters	Models				
	AdaBoost-EOA	GBRT-EOA	XGBRT-EOA	RF-EOA	Cubist-EOA
Number of estimators	188	98	73	58	–
Min_samples_split	0.0001	1e−6	–	3	–
Min_samples _leaf	0.043	7.719e−5	–	2	–
Max_depth	218	177	3	163	–
Max_features	6	4	–	5	–
Max_ leaf_nodes	400	483	52	108	–
Ccp_alpha	0.0	0.0	–	0.0	–
Min_weight_fraction_leaf	0.0309	0.0392	–	0.0006	–
Learning rate	0.008	0.1150	0.186	–	–
Alpha	–	0.547	–	–	–
Subsample	–	0.276	0.577	–	–
Max_samples	–	–	–	0.812	–
Number of rules	–	–	–	–	4552
Number of committees	–	–	–	–	3
Neighbors	–	–	–	–	4
Extrapolation	–	–	–	–	0.177
Gamma	–	–	0.0	–	–
Min_child_weight	–	–	0.983	–	–
Reg_lambda	–	–	1.509	–	–
Colsample_bytree	–	–	0.958	–	–

## Evaluation of model prediction performance

**Figure 13:**
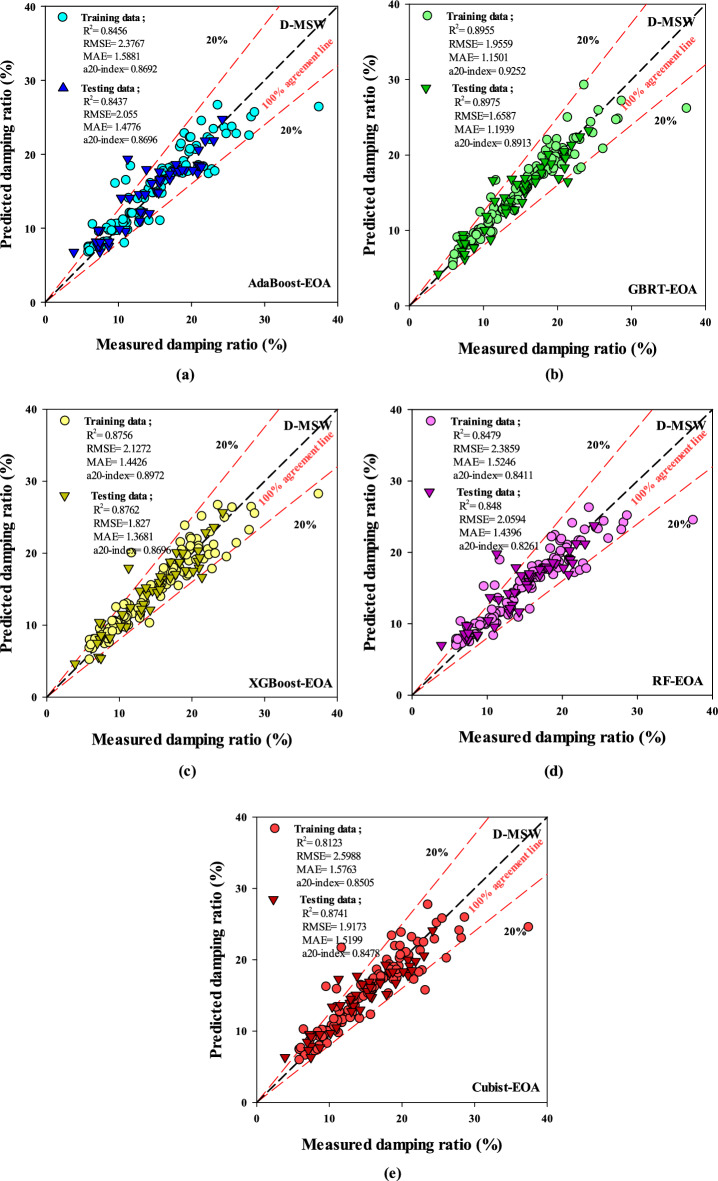
Measured versus expected scattering dots for D-MSW.

As depicted in Fig. 13, during the test and train phases, a higher R^2^ value and a lower degree of scattering points mean that the GBRT-EOA model outperforms other D-MSW models. Also, the low RMSE and MAE values of the introduced XGBoost-EOA model demonstrate its high accuracy and reliability in both training and testing  processes with a slight difference compared to GBRT-EOA. The A20-index is a newly developed physical engineering metric that measures the number of specimens in which estimated quantities deviate by at most 20% from observed values^60^. An a20-index = 0.9252 in the training phase and a20-index = 0.8913 in the testing phase make GBRT-EOA the top performer in predicting D-MSW.

Comparing the performance of various models on testing and training datasets reveals that EOA generally enhances the model accuracy (Table 6). For instance, GBRT-EOA exhibits the best performance on the testing set, with the highest R^2^ value (0.8975) and the lowest error metrics (MAPE = 0.0954, MAE = 1.1939, and RMSE = 1.6587), significantly outperforming its default counterpart (R^2^ = 0.8281, MAPE = 0.14461, MAE = 1.7976, and RMSE = 2.4059). Similarly, XGBoost-EOA and RF-EOA indicate substantial improvements in testing accuracy compared to their default versions, reflected in higher R^2^ values and lower error rates. Notably, XGBoost-Default achieves near-perfect performance on training data (R^2^ = 0.9996, MAPE = 0.0053, MAE = 0.0804,  and RMSE = 0.1168), but its testing performance (R^2^ = 0.6814, MAPE = 0.1597, MAE = 2.1399, and RMSE = 3.4910) suggests overfitting. AdaBoost-EOA also demonstrates significant improvement, with its testing R^2^ increasing from 0.6998 to 0.8437 and error metrics decreasing across the board compared to the default version. On the other hand, the Cubist model display less pronounced gains with EOA, revealing that this algorithm might be less responsive to enhanced optimization in this context. Overall, applying EOA leads to more reliable and accurate models, with GBRT-EOA standing out as the most robust, balancing high training accuracy with excellent generalization to unseen data. This comparison underscores the importance of optimization algorithms in refining model performance, particularly in complex prediction tasks.

**Table 6:** Precision and effectiveness of each EL model.

Models	Training				Testing			
	R^2^	RMSE	MAE	MAPE	R^2^	RMSE	MAE	MAPE
AdaBoost-Default	0.9959	0.3977	0.1434	0.0148	0.6998	3.5585	2.2178	0.1679
AdaBoost-EOA	0.8456	2.3767	1.5881	0.1093	0.8437	2.0550	1.4776	0.1269
GBRT- Default	0.8989	1.9111	1.2562	0.0824	0.8281	2.4059	1.7976	0.14461
GBRT-EOA	0.8955	1.9559	1.1501	0.0730	0.8975	1.6587	1.1939	0.0954
XGBoost- Default	0.9996	0.1168	0.0804	0.0053	0.6814	3.4910	2.1399	0.1597
XGBoost EOA	0.8756	2.1272	1.4426	0.0963	0.8762	1.8270	1.3681	0.1111
RF- Default	0.950	1.3964	0.8509	0.0559	0.7915	2.4634	1.7277	0.1366
RF-EOA	0.8479	2.3859	1.5246	0.1051	0.8480	2.0594	1.4396	0.1269
Cubist- Default	0.6781	3.4126	2.4825	0.1728	0.7703	2.4886	1.9092	0.1589
Cubist-EOA	0.8123	2.5988	1.5763	0.1059	0.8741	1.9173	1.5199	0.1290

**Figure 14:**
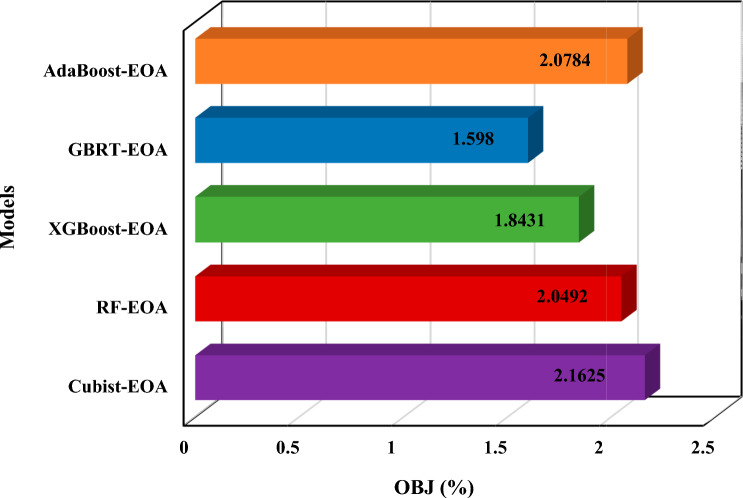
OBJ values of different proposed EL-EOA models.

Utilizing OBJ also makes it feasible to combine several statistical indicators for testing and training to evaluate a model’s performance^61^. High values of OBJ refer to the poor performance of a model compared to others^62^. According to Fig. 14, the GBRT-EOA model performs best with an OBJ of 1 for D-MSW. Also, regarding the OBJ values of 2.16% and 1.15%, the Cubist-EOA models are the worst performers for D-MSW.

As a further measure of the validity of the models, the Nash–Sutcliffe efficiency (NSE) coefficient, as well as the scatter index (SI), were used:

10 $$SI = \frac{RMSE}{{\overline{Y}_{obs} }}$$

11 $$NSE = 1 - \frac{{\sum\limits_{i = 1}^{N} {(Y_{pre} - Y_{obs} )^{2} } }}{{\sum\limits_{i = 1}^{N} {(Y_{obs} - \overline{Y}_{obs} )^{2} } }}$$

**Figure 15:**
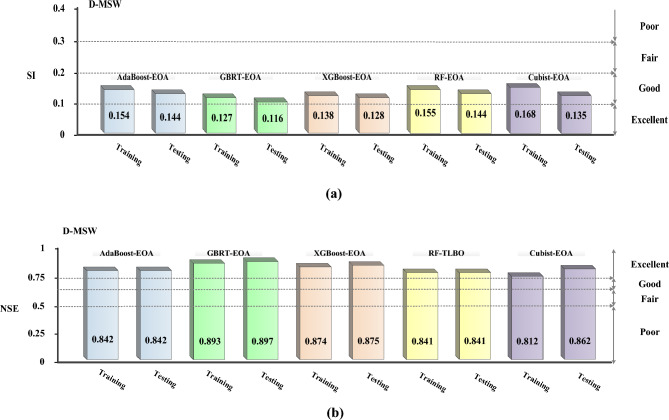
Evaluated SI and NSE values for each method.

In which the bar items over the associated digits represent the average of each value, *N* designates the records number, *Y*_*obs*_ refers to the observed, and *Y*_*pre*_ denotes anticipated quantities. A model’s predictive accuracy can be considered excellent if the NSE value exceeds 0.75 or the SI value is less than 0.1, and good if the NSE value is between 0.65 and 0.75 or the SI value is between 0.1 and 0.2. However, it is fair if the NSE value is between 0.5 and 0.65 or if the SI value is between 0.2 and 0.3^57,58^. As seen in Fig. 15, practically all methods have the SI rates less than 0.2, indicating that they are excellent or good predictors of D-MSW. The results also reveal that all the methods had NSE rates greater than 0.75, exhibiting the EL–EOA models’ "excellent" accuracy in predicting output values.

**Figure 16:**
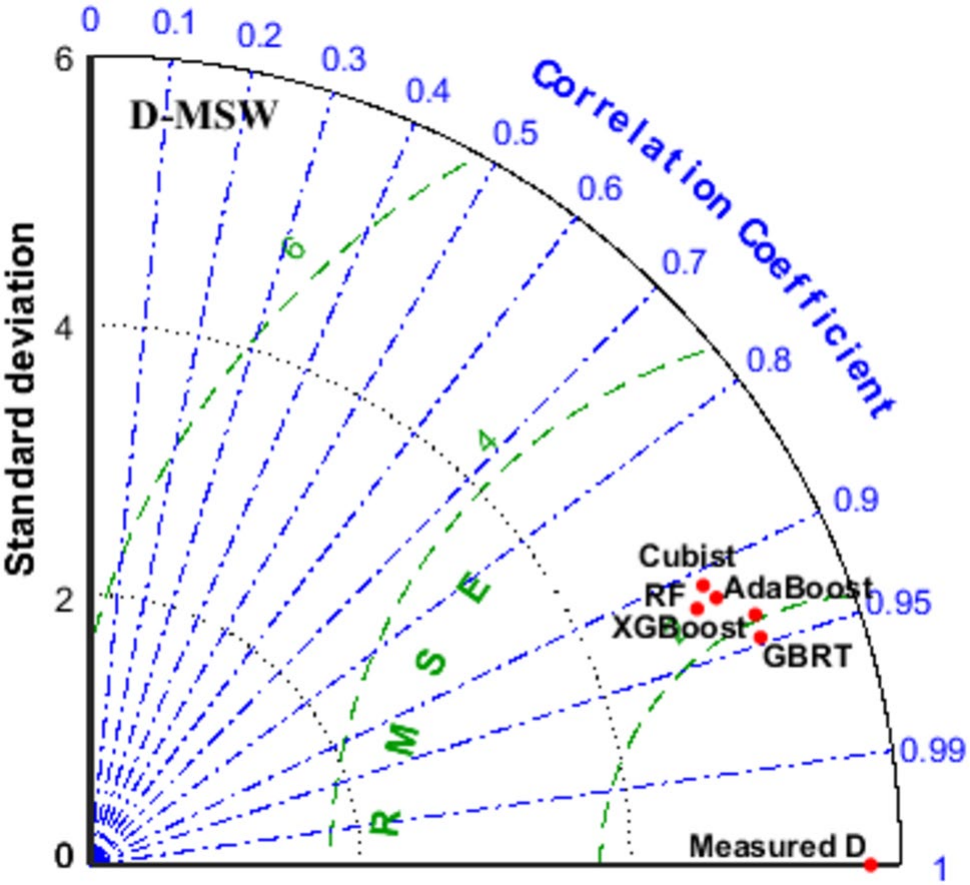
Taylor diagrams of various EL-EOA proposed models.

The effectiveness of each design was evaluated utilizing the diagram of Taylor presented in Fig. 16. To compare the anticipated outcomes with the actual values, three statistical measures of RMSE, standard deviation (STD), and R^2^ were applied. The standard deviation is depicted through a circle connecting the plot’s axes of horizontal and vertical; RMSE is illustrated by the horizontal green dots, and the blue line suggests the values of R^2^. In this regard, the GBRT-EOA models have the best performance among all the models for D-MSW. As shown in the figure, the furthest state belongs to the Cubist-EOA for D-MSW.

**Figure 17:**
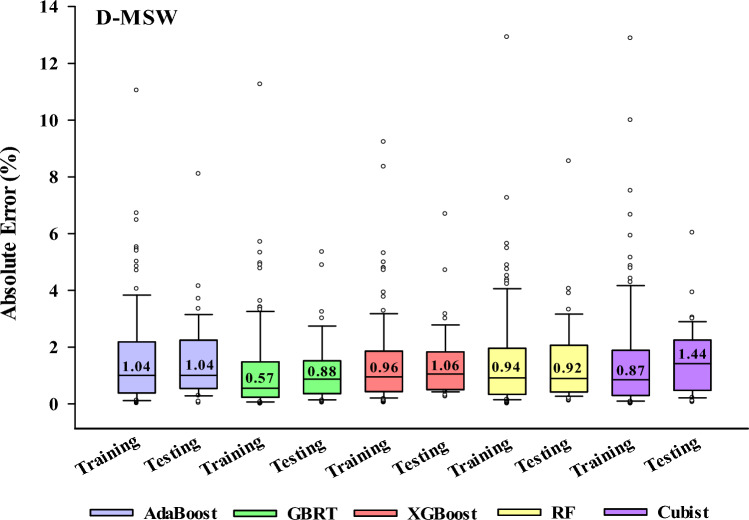
Absolute error boxes of various EL-EOA methods for both training and testing phases.

Figure 17 displays the absolute error boxes of all the models for the test and train datasets. GBRT-EOA has the least interquartile ranges in training phases for D-MSW, demonstrating this model’s outstanding capability. Furthermore, the median absolute error quantities of GBRT-EOA methods are 0.88 in the test stage for D-MSW, which indicates the decisive superiority of these models compared to others.

## Comparison of K-fold cross-validation and hold-out methods

In studies with relatively small datasets, like the current one, data were typically split into training and testing sets to control overfitting and enhance the prediction performance of the ML models. External validation, which uses datasets from various published sources, can be beneficial but was unavailable due to the novel nature of this research. Consequently, *K*-fold cross-validation (CV) is a viable alternative for assessing the model performance. This method randomly divides the training set into *K* equal-sized subsets, using *K* − 1 subsets for training and the remaining subset for validation. Here, a fivefold CV approach was implemented, allocating 70% of the initial dataset as the training set and 30% as the test set. The optimum hyper-parameters for the best performing model (GBRT), were determined using EOA to achieve the lowest average RMSE over the five training folds. Subsequently, these hyper-parameters were applied to train the GBRT model for D-MSW on the entire training dataset, and the model’s efficacy was evaluated on the testing dataset. Table 7 compares the performance of GBRT models utilizing both hold-out and *K*-fold CV methods, noting that RMSE and MAE in *K*-fold CV are based on normalized data. The optimal hyper-parameters derived from the *K*-fold CV for D-MSW are detailed in Table 8.

**Table 7:** Comparative statistical analysis of GBRT model for D-MSW using K-fold cross-validation and hold-out methods.

Folds	R^2^	RMSE	MAE	MAPE
K-1	0.8662	0.1099	0.0843	0.9154
K-2	0.7630	0.1642	0.1265	0.6704
K-3	0.8825	0.1083	0.0748	1.1665
K-4	0.5917	0.2924	0.1769	1.5284
K-5	0.7626	0.1634	0.1178	3.0403
Mean folds	0.7732	0.1676	0.1161	1.4642
Training-K-folds	0.7854	2.7733	1.7680	0.1220
Testing-K-folds	0.7973	2.3705	1.5706	0.1342
Training-hold out	0.8955	1.9559	1.1501	0.0730
Testing-hold out	0.8975	1.6587	1.1939	0.0954

**Table 8:** Optimal hyper parameters for GBRT model of D-MSW determined via K-fold cross-validation.

Parameters	Optimal values
Learning_rate	1.1846
N_estimators	148
Subsample	0.9849
Min_samples_split	0.6775
Min_samples_leaf	1.0214
Min_weight_fraction_leaf	0.2219
Max_depth	11
Max_features	5
Max_leaf_nodes	89
Alpha	0.3329
Ccp_alpha	0

## SHAP study

**Figure 18:**
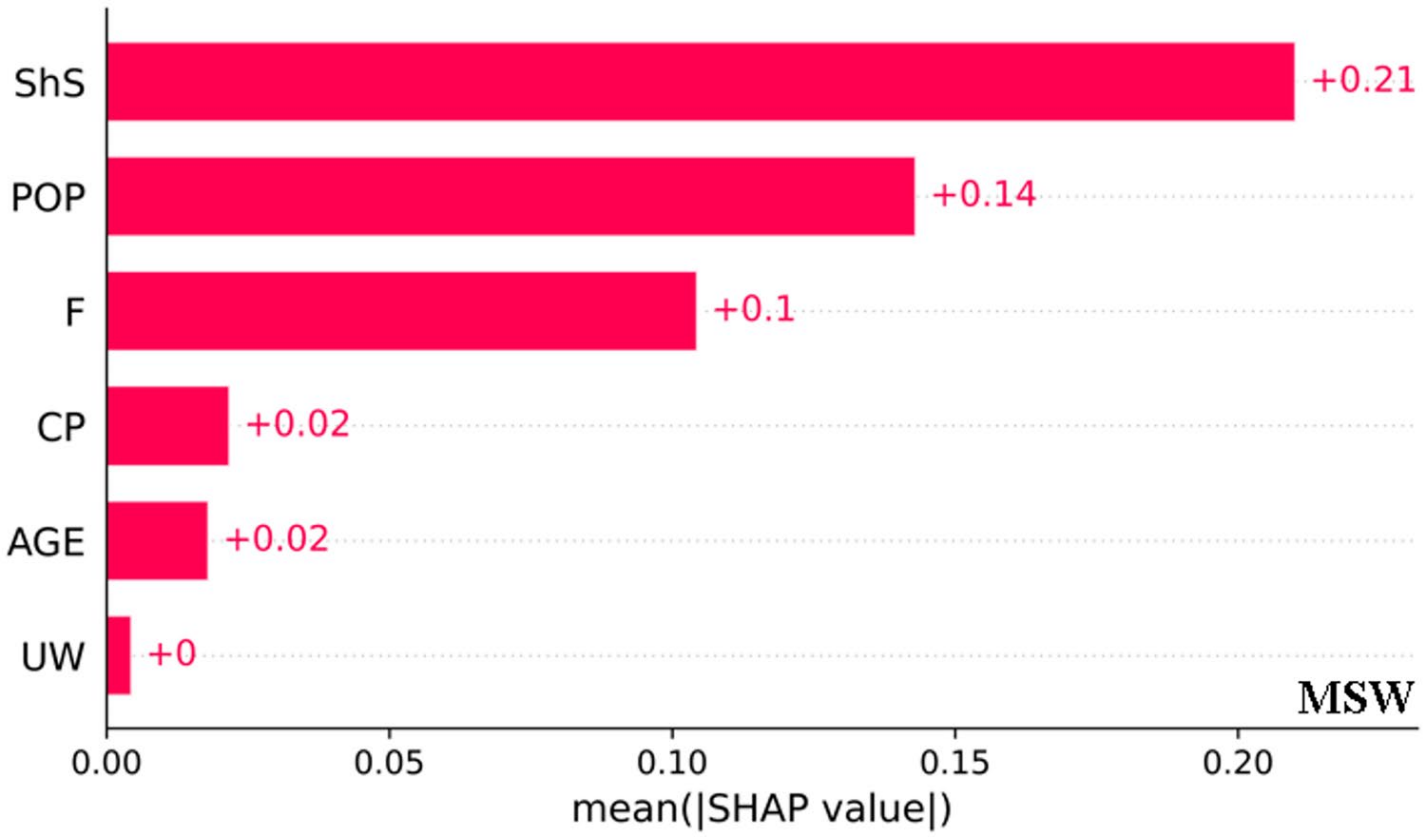
Feature significance of input variables.

SHAP is a game-theoretic approach designed to describe the result of machine-learning methods^63^. SHAP presents the features’ contribution to the model’s output, offering a more interpretable and transparent understanding of the model’s decision-making process. In the ensuing sections, we thoroughly analyze the outcomes in the proposed predicting structure, designed to interpret and comprehend the results of the probabilistic predicting model. Our primary focus is examining how the developed model utilizes various features to make predictions. The SHAP method is employed for explanations, covering D-MSW. Figure 18 illustrates the average contribution of each feature, with each bar plot representing the importance of a specific property. Shear strain plays a significant role in the MSW (+ 0.21) model, contributing more substantially. *Age* and *UW* (total + 0.02) for MSW exhibit minimal impact on the output of the models.

**Figure 19:**
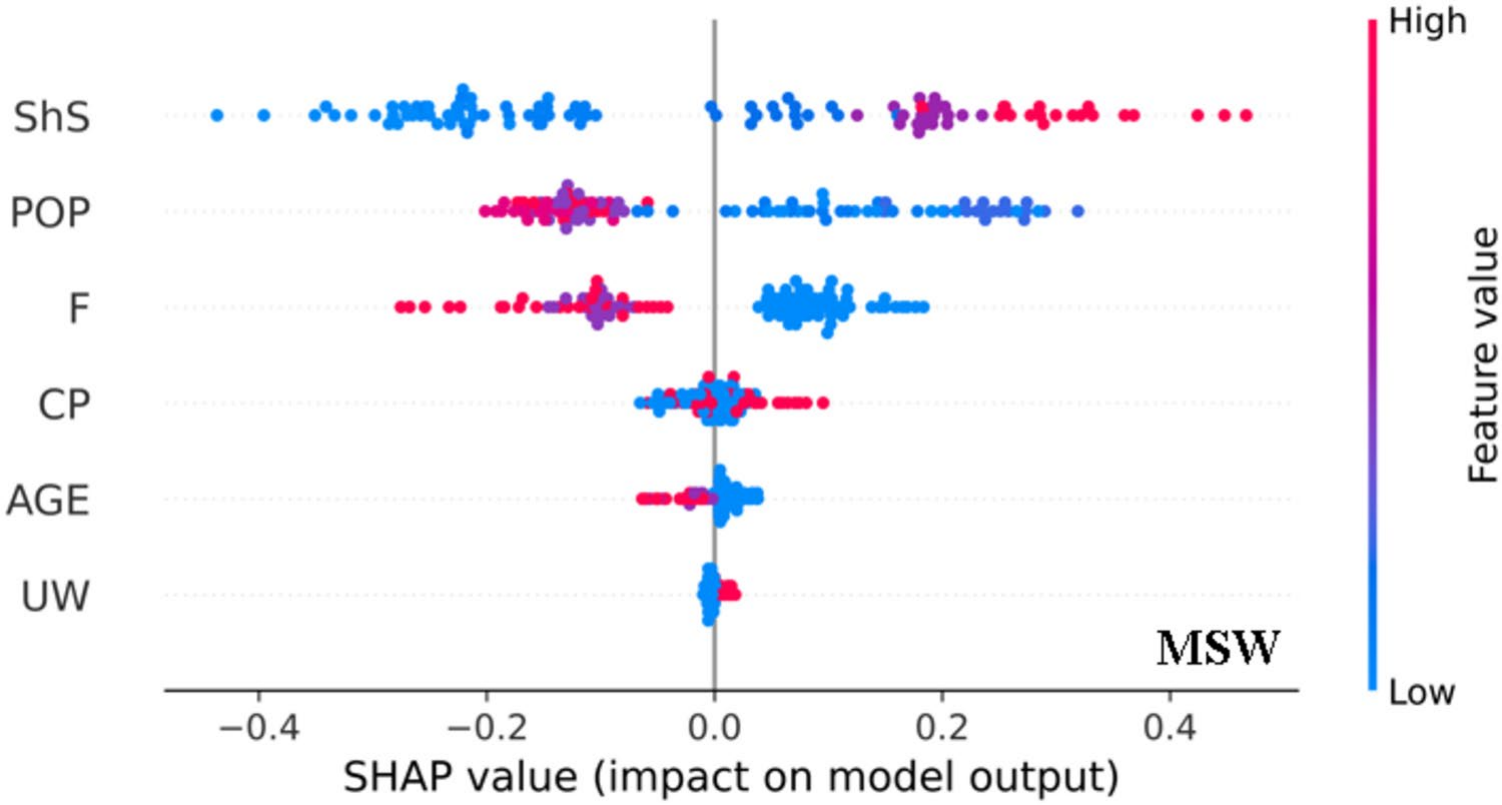
Summary plot of point predicting model.

Each dot in Fig. 19 represents a distinct predicting, and its location along the x-axis signifies the impact of that attribute on the output of the model. Furthermore, each dot’s color corresponds to a feature value (varies from blue to red), emphasizing the relative contributions of different feature values to the result. Long tails show highly significant characteristics. The dots’ vertical distribution suggests more findings with comparable effects. These SHAP summary graphs in such a setting include details on the number of reports that have those qualities as well as the size and direction of each feature’s effect. For example, elevated shear strain values (in MSW models) tend to elevate the model output, while values closer to zero for shear strain led to decreased model output (for MSW model). Therefore, the impact on the model output becomes more substantial with higher shear strain values in MSW.

**Figure 20:**
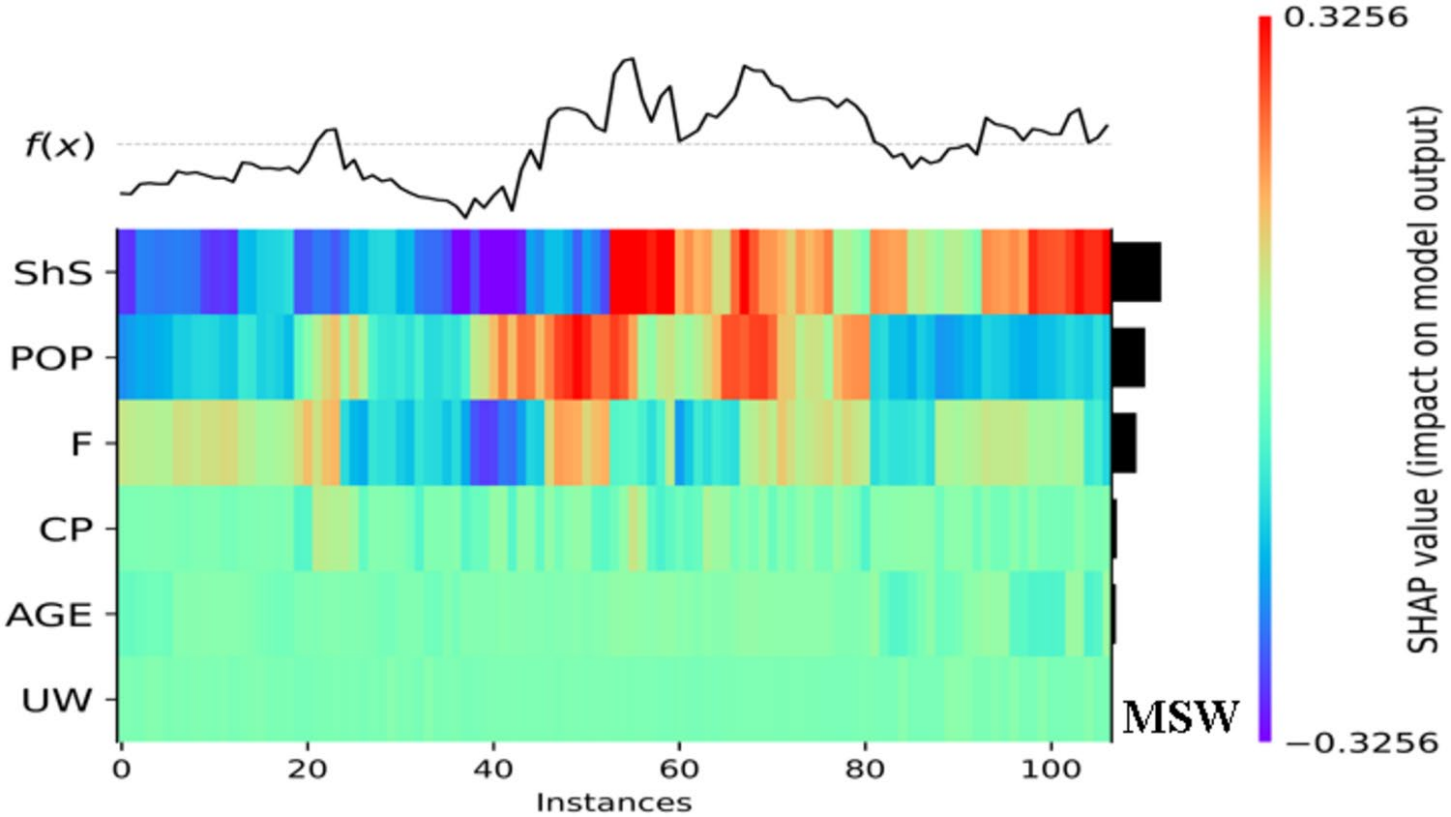
SHAP values and function variations of each target.

In the following, the function of variations for each output is presented in Fig. 20. The ranges of the SHAP values, which refer to the impact on each model target, are depicted by various colors ranging from blue to red. For instance, the function of MSW variations is increasing and decreasing at lower instances which is highly dependent on the *ShS* and *PoP* values.

## Online application of proposed D-MSW models

**Figure 21:**
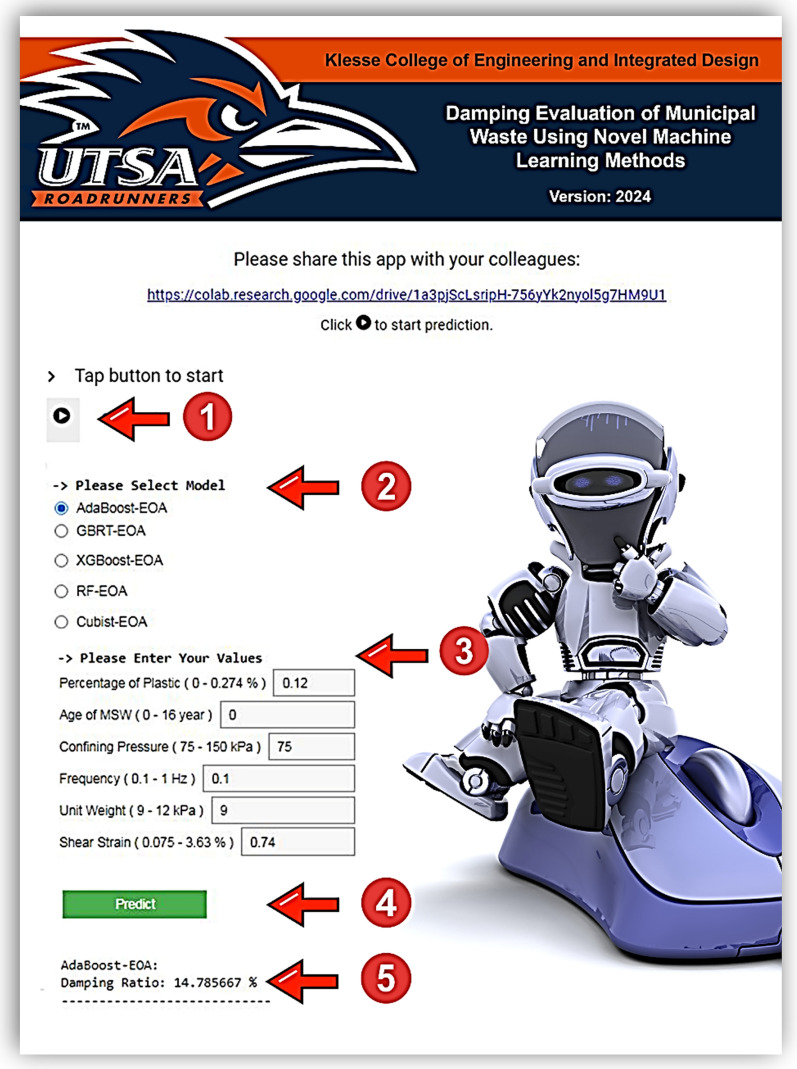
Online application of proposed D-MSW models.

Models developed using EL-FOA methods differ from classical regression methods as they do not simply relate inputs and outputs^64^. In this regard, implementing a Python-based online application makes it possible for researchers and practicing engineers, the end users of the proposed D-MSW models, to estimate values of dynamic properties easily. In the past, several researchers have developed software using MATLAB graphical user interface (GUI) to predict the properties of different materials^65,66^. There are several advantages to the developed online application: (i) results are available more quickly, and it provides a standard for an in-depth investigation of mix designs; (ii) reducing production costs while ensuring the safety and quality concerns as well, it allows to determine if a mix design is reasonable; and (iii) in addition to being easy to use, it will reduce human errors in calculations^67^. Free access to this online application is also provided for all researchers^68^. This application enables engineers and researchers to obtain a relatively accurate prediction of D-MSW at their project site in simple steps, as demonstrated in Fig. 20. To clarify, assume that PoP, Age, CP, F, UW, and ShS, as highlighted in Fig. 21, are equal to 0.12%, 0 year, 75 kPa, 0.1 Hz, 9 kPa, and 0.74%, respectively. First, the user simply needs to click on the run code button in the left corner. To continue the predicting process, the desired model type is chosen in the second step. Next, the assumed input values are entered in the embedded boxes next to each parameter. Users are guided by the max and min values of each input variable. These values are included to prevent errors in prediction since the dataset that trained the models was in the same range. Upon clicking the predict button, the result is displayed. The user can then view a new result and compare it with the previous results by changing the model type and inputs.

## Summary and conclusions

Landfill stabilization under seismic loading is dependent on the components, including MSW. To achieve sustainable design and development of landfills, D is regarded as a crucial characteristic that should be assessed. A better waste management procedure may be established by determining the factors influencing the seismic performance of landfills. In this study, a novel dataset derived from laboratory experiments was utilized to develop predictive models for the damping response of landfills, employing innovative AI techniques. For this reason, 153 cyclic triaxial tests were performed on MSW. Following that, five AI-based methods including AdaBoost-EOA, GBRT-EOA, XGBoost-EOA, RF-EOA, and Cubist-EOA were adopted to formulate the produced laboratory damping data. A summary of the study’s findings is provided below:

- The correlation heatmap analysis revealed that D-MSW in datasets was more dependent on shear strain (+ 0.62). Meanwhile, the vertical load (− 0.41) and percentage of plastic (− 0.33) had the most minor correlations with the target in the D-MSW datasets, respectively.
- The investigation of the performance parameters and comparison of various EL models indicated that the GBRT-EOA models presented better performance than the other methods for D-MSW. The OBJ value (which exhibits the model’s performance) of the aforementioned best model (GBRT-EOA) was 1.6%. Also, regarding the OBJ value of 2.16%, the Cubist-EOA model was the worst performer for D-MSW.
- The SHAP examination of the input factors demonstrated that a significant share of D-MSW came from shear strain changes. However, the unit weight for D-MSW (0)  had the most negligible impact on the modeling procedure.

## Data Availability

Data associated with the present study will be available on request from the corresponding authors.

## Abbreviations

R^2^: Coefficient of determination
AdaBoost: Adaptive boosting
AI: Artificial intelligence
ANFIS: Adaptive neuro-fuzzy inference system
ANN: Artificial neural network
BPNN: Back-propagation neural network
CP: Confining pressure
CRRF: Classification and regression random forest
D: Damping
EOA: Equilibrium optimizer algorithm
F: Frequency
FNN: Feed forward neural network
G: Shear modulus
GBRT: Gradient boosting regression tree
GEP: Gene expression programming
LSSVM: Least square support vector machine
LSTM: Long short-term memory
MAE: Mean absolute error
MAPE: Mean absolute percentage error
MARS: Multivariate adaptive regression spline
ML: Machine learning
MLP: Multilayer perceptron
MRM: Multiple regression analysis method
NSE: Nash–Sutcliffe efficiency
PoP: Percentage of plastic
RF: Random forest
RMSE: Root mean square error
ShS: Shear strain
SI: Scatter index
STD: Standard deviation
SVM: Support vector machine
SVR: Support vector regression
USCS: Unified soil classification system
UW: Unit weight
VL: Vertical load
XGBoost: Extreme gradient boosting

## References

[1] Kaza, S., Yao, L., Bhada-Tata, P. & Van Woerden, F. *What a Waste 20: A Global Snapshot of Solid Waste Management to 2050* (World Bank Publications, 2018).

[2] Alidoust, P., Kargar, P., Goodarzi, S., Keramati, M. & Moradi Moqaddam, H. Laboratory-based assessment on similarities between dynamic behavior of MSW and clay. *J. Mater. Cycles Waste Manage.* **23**, 622–643 (2021). 10.1007/s10163-020-01151-x

[3] Keramati, M., Goodarzi, S., Moradi Moghadam, H. & Ramesh, A. Evaluating the stress–strain behavior of MSW with landfill aging. *Int. J. Environ. Sci. Technol.* **16**, 6885–6894 (2019). 10.1007/s13762-018-2106-z

[4] Karimpour-Fard, M. *et al.* Laboratory study on dynamic properties of municipal solid waste in Saravan Landfill, Iran. *Int. J. Civ. Eng.* **19**, 861–879 (2021). 10.1007/s40999-020-00588-3

[5] Rawat, P. & Mohanty, S. Parametric study on dynamic characterization of municipal solid waste fine fractions for geotechnical purpose. *J. Hazard. Toxic Radioact. Waste* **26**(1), 04021047 (2022). 10.1061/(ASCE)HZ.2153-5515.0000659

[6] Akbarimehr, D. & Fakharian, K. Dynamic shear modulus and damping ratio of clay mixed with waste rubber using cyclic triaxial apparatus. *Soil Dyn. Earthq. Eng.* **140**, 106435 (2021). 10.1016/j.soildyn.2020.106435

[7] Patiño, H. & Galindo, R. Damping of a soft marine clay subjected to the combination of monotonic and cyclic shear stresses. *Eur. J. Environ. Civ. Eng.* **27**(1), 72–95 (2023). 10.1080/19648189.2022.2030804

[8] Abdellaziz, M. *et al.* Shear modulus and hysteretic damping of sensitive eastern Canada clays. *Can. Geotech. J.* **58**(8), 1118–1134 (2021). 10.1139/cgj-2020-0254

[9] Alidoust, P., Keramati, M. & Shariatmadari, N. Laboratory studies on effect of fiber content on dynamic characteristics of municipal solid waste. *Waste Manage.* **76**, 126–137 (2018). 10.1016/j.wasman.2018.02.038 29500081

[10] Amlashi, A. T., Abdollahi, S. M., Goodarzi, S. & Ghanizadeh, A. R. Soft computing based formulations for slump, compressive strength, and elastic modulus of bentonite plastic concrete. *J. Clean. Prod.* **230**, 1197–1216 (2019). 10.1016/j.jclepro.2019.05.168

[11] Bagheri, M., Esfilar, R., Golchi, M. S. & Kennedy, C. A. A comparative data mining approach for the prediction of energy recovery potential from various municipal solid waste. *Renew. Sustain. Energy Rev.* **116**, 109423 (2019). 10.1016/j.rser.2019.109423

[12] Liang, R. *et al.* Interpretable machine learning assisted spectroscopy for fast characterization of biomass and waste. *Waste Manage.* **160**, 90–100 (2023). 10.1016/j.wasman.2023.02.012 36801592

[13] Khatti, J. & Grover, K. S. Assessment of fine-grained soil compaction parameters using advanced soft computing techniques. *Arab. J. Geosci.* **16**(3), 208 (2023). 10.1007/s12517-023-11268-6

[14] Zhang, Y., Liu, J. & Shen, W. A review of ensemble learning algorithms used in remote sensing applications. *Appl. Sci.* **12**(17), 8654 (2022). 10.3390/app12178654

[15] Chen, C. H., Tanaka, K., Kotera, M. & Funatsu, K. Comparison and improvement of the predictability and interpretability with ensemble learning models in QSPR applications. *J. Cheminform.* **12**, 1–16 (2020). 33430997 10.1186/s13321-020-0417-9 PMC7106596

[16] Moradi Moghaddam, H. *et al.* Shear modulus prediction of landfill components using novel machine learners hybridized with forensic-based investigation optimization. *Constr. Build. Mater.* **411**, 134443 (2024). 10.1016/j.conbuildmat.2023.134443

[17] Ahmad, M. *et al.* Extreme gradient boosting algorithm for predicting shear strengths of rockfill materials. *Complexity* **2022**, 1–11 (2022). 10.1155/2022/9415863

[18] Choudhury, D. & Savoikar, P. Equivalent-linear seismic analyses of MSW landfills using DEEPSOIL. *Eng. Geol.* **107**(3–4), 98–108 (2009). 10.1016/j.enggeo.2009.05.004

[19] Keramati, M., Moradi Moqhaddam, H., Mozaffari, O. & Fayeghi, M. Evaluation of the effects of aging and different site conditions on the seismic response of municipal solid waste (A Case of Kahrizak Landfill). *AUT J. Civ. Eng.* **5**(3), 377–388 (2021).

[20] Faramarzi, A., Heidarinejad, M., Stephens, B. & Mirjalili, S. Equilibrium optimizer: A novel optimization algorithm. *Knowl.-Based Syst.* **191**, 105190 (2020). 10.1016/j.knosys.2019.105190

[21] Elgamal, Z. M. *et al.* Improved equilibrium optimization algorithm using elite opposition-based learning and new local search strategy for feature selection in medical datasets. *Computation* **9**(6), 68 (2021). 10.3390/computation9060068

[22] Ghanizadeh, A. R. *et al.* A comparison of novel hybrid ensemble learners to predict the compressive strength of green engineering materials: a case of concrete composed of rice husk ash. *Eur. J. Environ. Civ. Eng.*10.1080/19648189.2024.2335343 (2024). 10.1080/19648189.2024.2335343

[23] Ghanizadeh, A. R. *et al.* Intelligent prediction of asphalt concrete air voids during service life using cubist and GBRT-ensemble learning approaches hybridized with an equilibrium optimizer algorithm. *J. Mater. Civ. Eng.* **36**(5), 04024098 (2024). 10.1061/JMCEE7.MTENG-17222

[24] Alidoust, P. *et al.* Prediction of the shear modulus of municipal solid waste (MSW): An application of machine learning techniques. *J. Clean. Prod.* **303**, 127053 (2021). 10.1016/j.jclepro.2021.127053

[25] Gatto, M. P. A. & Montrasio, L. Artificial Neural Network model to predict the dynamic properties of sand-polyurethane composite materials for GSI applications. *Soil Dyn. Earthq. Eng.* **172**, 108032 (2023). 10.1016/j.soildyn.2023.108032

[26] Wu, Q., Wang, Z., Qin, Y. & Yang, W. Intelligent model for dynamic shear modulus and damping ratio of undisturbed marine clay based on Back-Propagation neural network. *J. Mar. Sci. Eng.* **11**(2), 249 (2023). 10.3390/jmse11020249

[27] Baghbani, A., Choudhury, T., Samui, P. & Costa, S. Prediction of secant shear modulus and damping ratio for an extremely dilative silica sand based on machine learning techniques. *Soil Dyn. Earthq. Eng.* **165**, 107708 (2023). 10.1016/j.soildyn.2022.107708

[28] Baghbani, A., Costa, S., Faradonbeh, R. S., Soltani, A. & Baghbani, H. Modeling the effects of particle shape on damping ratio of dry sand by simple shear testing and artificial intelligence. *Appl. Sci.* **13**(7), 4363 (2023). 10.3390/app13074363

[29] Pasha, S. M. K., Hazarika, H. & Yoshimoto, N. Estimating dynamic characteristics of gravel-tire chips mixtures using artificial intelligence techniques. *J. Scoc. Mater. Sci. (材料)* **69**(1), 1–8 (2020).

[30] Keshavarz, A. & Mehramiri, M. New Gene Expression Programming models for normalized shear modulus and damping ratio of sands. *Eng. Appl. Artif. Intell.* **45**, 464–472 (2015). 10.1016/j.engappai.2015.07.022

[31] Javdanian, H., Jafarian, Y. & Haddad, A. Predicting damping ratio of fine-grained soils using soft computing methodology. *Arab. J. Geosci.* **8**, 3959–3969 (2015). 10.1007/s12517-014-1493-9

[32] Edincliler, A., Cabalar, A. F. & Cevik, A. Modelling dynamic behaviour of sand–waste tires mixtures using Neural Networks and Neuro-Fuzzy. *Eur. J. Environ. Civ. Eng.* **17**(8), 720–741 (2013). 10.1080/19648189.2013.814552

[33] Samui, P. & Kothari, D. P. A multivariate adaptive regression spline approach for prediction of maximum shear modulus and minimum damping ratio. *Eng. J.* **16**(5), 69–78 (2012). 10.4186/ej.2012.16.5.69

[34] Cevik, A. & Cabalar, A. F. Modelling damping ratio and shear modulus of sand–mica mixtures using genetic programming. *Expert Syst. Appl.* **36**(4), 7749–7757 (2009). 10.1016/j.eswa.2008.09.010

[35] Cabalar, A. F. & Cevik, A. Modelling damping ratio and shear modulus of sand–mica mixtures using neural networks. *Eng. Geol.* **104**(1–2), 31–40 (2009). 10.1016/j.enggeo.2008.08.005

[36] Akbulut, S., Hasiloglu, A. S. & Pamukcu, S. Data generation for shear modulus and damping ratio in reinforced sands using adaptive neuro-fuzzy inference system. *Soil Dyn. Earthq. Eng.* **24**(11), 805–814 (2004). 10.1016/j.soildyn.2004.04.006

[37] Keramati, M., Moghaddam, H. M. & Ramesh, A. Prediction of the stress-strain behavior of MSW materials using Hyperbolic model and Evolutionary Polynomial Regression (EPR). *Amirkabir J. Civ. Eng.* **51**(4), 793–804 (2019).

[38] Zekkos, D. P. *et al*. Framework for the estimation of MSW unit weight profile. *Proc., 10th Int. Waste Management and Landfill Symp*.

[39] Pintelas, E., Livieris, I. E. & Pintelas, P. A grey-box ensemble model exploiting black-box accuracy and white-box intrinsic interpretability. *Algorithms* **13**(1), 17 (2020). 10.3390/a13010017

[40] Schapire, R. E. Explaining adaboost. *Empirical Inference: Festschrift in Honor of Vladimir N. Vapnik* 37–52 (2013).

[41] Prettenhofer, P. & Louppe, G. Gradient boosted regression trees in scikit-learn. *PyData* (2014).

[42] Chen, T. *et al.* Xgboost: extreme gradient boosting. *R Package Version* **1**(4), 1–4 (2015).

[43] Zhao, X. Y., Chen, J. X., Chen, G. M., Xu, J. J. & Zhang, L. W. Prediction of ultimate condition of FRP-confined recycled aggregate concrete using a hybrid boosting model enriched with tabular generative adversarial networks. *Thin Walled Struct.* **182**, 110318 (2023). 10.1016/j.tws.2022.110318

[44] Svetnik, V. *et al.* Random forest: a classification and regression tool for compound classification and QSAR modeling. *J. Chem. Inf. Comput. Sci.* **43**(6), 1947–1958 (2003). 14632445 10.1021/ci034160g

[45] Zhang, J., Ma, G., Huang, Y., Aslani, F. & Nener, B. Modelling uniaxial compressive strength of lightweight self-compacting concrete using random forest regression. *Constr. Build. Mater.* **210**, 713–719 (2019). 10.1016/j.conbuildmat.2019.03.189

[46] Breiman, L. Random forests. *Mach. Learn.* **45**, 5–32 (2001). 10.1023/A:1010933404324

[47] Quinlan, J. R. *Learning with Continuous Classes*. In *5th Australian Joint Conference on Artificial Intelligence* (World Scientific, 1992).

[48] Houborg, R. & McCabe, M. F. A hybrid training approach for leaf area index estimation via Cubist and random forests machine-learning. *ISPRS J. Photogramm. Remote Sens.* **135**, 173–188 (2018). 10.1016/j.isprsjprs.2017.10.004

[49] Rulequest. *Data Mining with Cubist* (2016).

[50] Wang, Y. & Witten, I. H. *Induction of Model Trees for Predicting Continuous Classes* (1996).

[51] Quinlan, J. R. Combining instance-based and model-based learning. *Proceedings of the Tenth International Conference on Machine Learning* (1993).

[52] Zhou, J. *et al.* Random forests and cubist algorithms for predicting shear strengths of rockfill materials. *Appl. Sci.* **9**(8), 1621 (2019). 10.3390/app9081621

[53] Xu, Y. *et al.* Evaluation of machine learning techniques with multiple remote sensing datasets in estimating monthly concentrations of ground-level PM2. 5. *Environ. Pollut.* **242**, 1417–1426 (2018). 30142557 10.1016/j.envpol.2018.08.029

[54] Lee, D. G. & Ahn, K. H. “A stacking ensemble model for hydrological post-processing to improve streamflow forecasts at medium-range timescales over South Korea. *J. Hydrol.* **600**, 126681 (2021). 10.1016/j.jhydrol.2021.126681

[55] Shaheen, A. M., Elsayed, A. M., El-Sehiemy, R. A. & Abdelaziz, A. Y. Equilibrium optimization algorithm for network reconfiguration and distributed generation allocation in power systems. *Appl. Soft Comput.* **98**, 106867 (2021). 10.1016/j.asoc.2020.106867

[56] Iqbal, M. F. *et al.* Prediction of mechanical properties of green concrete incorporating waste foundry sand based on gene expression programming. *J. Hazard. Mater.* **384**, 121322 (2020). 31604206 10.1016/j.jhazmat.2019.121322

[57] Tavana Amlashi, A., Mohammadi Golafshani, E., Ebrahimi, S. A. & Behnood, A. Estimation of the compressive strength of green concretes containing rice husk ash: a comparison of different machine learning approaches. *Eur. J. Environ. Civ. Eng.* **27**(2), 961–983 (2022). 10.1080/19648189.2022.2068657

[58] Alidoust, P., Goodarzi, S., Tavana Amlashi, A. & Sadowski, Ł. Comparative analysis of soft computing techniques in predicting the compressive and tensile strength of seashell containing concrete. *Eur. J. Environ. Civ. Eng.* **27**, 1853–1875 (2022). 10.1080/19648189.2022.2102081

[59] Sadaghat, B. *et al.* Evaluating strength properties of Eco-friendly Seashell-Containing Concrete: Comparative analysis of hybrid and ensemble boosting methods based on environmental effects of seashell usage. *Eng. Appl. Artif. Intell.* **133**, 108388 (2024). 10.1016/j.engappai.2024.108388

[60] Asteris, P. G., Ashrafian, A. & Rezaie-Balf, M. Prediction of the compressive strength of self-compacting concrete using surrogate models. *Comput. Concr.* **24**(2), 137–150 (2019).

[61] Golafshani, E. M. & Behnood, A. Predicting the mechanical properties of sustainable concrete containing waste foundry sand using multi-objective ANN approach. *Constr. Build. Mater.* **291**, 123314 (2021). 10.1016/j.conbuildmat.2021.123314

[62] Ashrafian, A., Gandomi, A. H., Rezaie-Balf, M. & Emadi, M. An evolutionary approach to formulate the compressive strength of roller compacted concrete pavement. *Measurement* **152**, 107309 (2020). 10.1016/j.measurement.2019.107309

[63] Lundberg, S. M. & Lee, S. I. A unified approach to interpreting model predictions. *Adv. Neural Inf. Process. Syst.* **30**, 4768–4777 (2017).

[64] Heidarabadizadeh, N., Ghanizadeh, A. R. & Behnood, A. Prediction of the resilient modulus of non-cohesive subgrade soils and unbound subbase materials using a hybrid support vector machine method and colliding bodies optimization algorithm. *Constr. Build. Mater.* **275**, 122140 (2021). 10.1016/j.conbuildmat.2020.122140

[65] Golafshani, E. M. & Behnood, A. Application of soft computing methods for predicting the elastic modulus of recycled aggregate concrete. *J. Clean. Prod.* **176**, 1163–1176 (2018). 10.1016/j.jclepro.2017.11.186

[66] Golafshani, E. M. & Behnood, A. Estimating the optimal mix design of silica fume concrete using biogeography-based programming. *Cement Concret. Compos.* **96**, 95–105 (2019). 10.1016/j.cemconcomp.2018.11.005

[67] Chou, J. S., Chen, L. Y. & Liu, C. Y. Forensic-based investigation-optimized extreme gradient boosting system for predicting compressive strength of ready-mixed concrete. *J. Comput. Des. Eng.* **10**(1), 425–445 (2023).

[68] D-MSW Web Application https://colab.research.google.com/drive/15aMqMcLCk_288jApPgjvRikd2S7bGZVP?usp=sharing (2024).

